# Association between Variants of the Leptin Receptor Gene (*LEPR*) and Overweight: A Systematic Review and an Analysis of the CoLaus Study

**DOI:** 10.1371/journal.pone.0026157

**Published:** 2011-10-18

**Authors:** Nicole Bender, Noëmi Allemann, Diana Marek, Peter Vollenweider, Gérard Waeber, Vincent Mooser, Matthias Egger, Murielle Bochud

**Affiliations:** 1 Institute of Social and Preventive Medicine, University of Bern, Bern, Switzerland; 2 Institute for Human Evolution, University of the Witwatersrand, Johannesburg, South Africa; 3 Department of Medical Genetics, Swiss Institute of Bioinformatics, University of Lausanne, Switzerland, Lausanne, Switzerland; 4 Department of Medicine, Centre Hospitalier Universitaire Vaudois, Lausanne, Switzerland; 5 GlaxoSmithKline, Philadelphia, Pennsylvania, United States of America; 6 Institute of Social and Preventive Medicine, Centre Hospitalier Universitaire Vaudois, University of Lausanne, Lausanne, Switzerland; I2MC INSERM UMR U1048, France

## Abstract

**Background:**

Three non-synonymous single nucleotide polymorphisms (Q223R, K109R and K656N) of the leptin receptor gene (*LEPR*) have been tested for association with obesity-related outcomes in multiple studies, showing inconclusive results. We performed a systematic review and meta-analysis on the association of the three *LEPR* variants with BMI. In addition, we analysed 15 SNPs within the *LEPR* gene in the CoLaus study, assessing the interaction of the variants with sex.

**Methodology/Principal Findings:**

We searched electronic databases, including population-based studies that investigated the association between *LEPR* variants Q223R, K109R and K656N and obesity- related phenotypes in healthy, unrelated subjects. We furthermore performed meta-analyses of the genotype and allele frequencies in case-control studies. Results were stratified by SNP and by potential effect modifiers. CoLaus data were analysed by logistic and linear regressions and tested for interaction with sex. The meta-analysis of published data did not show an overall association between any of the tested *LEPR* variants and overweight. However, the choice of a BMI cut-off value to distinguish cases from controls was crucial to explain heterogeneity in Q223R. Differences in allele frequencies across ethnic groups are compatible with natural selection of derived alleles in Q223R and K109R and of the ancient allele in K656N in Asians. In CoLaus, the rs10128072, rs3790438 and rs3790437 variants showed interaction with sex for their association with overweight, waist circumference and fat mass in linear regressions.

**Conclusions:**

Our systematic review and analysis of primary data from the CoLaus study did not show an overall association between *LEPR* SNPs and overweight. Most studies were underpowered to detect small effect sizes. A potential effect modification by sex, population stratification, as well as the role of natural selection should be addressed in future genetic association studies.

## Introduction

In the past few decades, the prevalence and incidence of obesity has rapidly increased globally and has reached epidemic proportions [1], [2]. Obesity is associated with many deleterious outcomes such as type 2 diabetes, hypercholesterolemia, hypertension or heart disease, and is directly related to increased mortality and reduced life expectancy [2]. With the completion of the human genome project and the first extensive genome-wide association studies, increasing numbers of risk alleles associated with obesity have been identified [3]–[5], some of them in genes not previously known to be associated with adiposity. However, to date, genome-wide association studies have only captured a small percentage of the genetic variance related to obesity or body mass index [6].

One commonly studied candidate gene for obesity, the leptin receptor gene (*LEPR*), is on a biologic pathway to obesity (leptin-insulin pathway) [7]. Leptin is produced in adipose tissue and in other organs. It is known to have pleiotropic actions, including regulation of several neuropeptides involved in appetite control [8] and thermogenesis [9]. Three non-synonymous single nucleotide polymorphisms of the *LEPR* gene (Q223R, K109R and K656N) have been tested for association with obesity-related outcomes in multiple studies, producing inconclusive results [e.g. 10], [11], [12], [13]. Two systematic reviews on these variants did not show an overall statistically significant association to obesity-related outcomes [14], [15]. However, many further studies on the association between *LEPR* variants and obesity have been published since the last review in 2005 [15], including studies on the interaction of these variants with sex or with other factors [e.g. 16]. We therefore performed a systematic review and meta-analysis on the association between the three *LEPR* variants Q223R, K109R and K656N and obesity-related outcomes.

As most studies do not report data stratified by sex or other possible effect modifiers, we additionally analysed 38 SNPs within the *LEPR* gene (among them K109R) in a cohort of more than 6000 Caucasian adults from the Swiss CoLaus study [17], (data unpublished to date). We assessed the association of the SNPs with a number of different obesity-related outcomes and for possible confounding variables, or interaction with sex. This approach allowed us to account for the need for more precise phenotyping of study subjects [18]. Furthermore, the issue of sample size in association studies is crucial [6]. Our approach provides data on a cohort with a larger sample size than the ones assessed in the systematic review.

## Materials and Methods

### Systematic review

We followed the guidelines PRISMA for the reporting of systematic reviews and meta-analyses [19]. The electronic databases Medline, Embase and ISI Web of Knowledge were searched (search date: 14 October 2009). The search strategy was carried out for all exposures and outcomes of interest. Search terms included *LEPR*, leptin receptor gene, Q223R, K109R, K656N, obesity, body mass index, BMI, weight, waist, waist-to-hip ratio, WHR, body fat, adiposity, overweight, fat mass, Quetelet index. Where possible, MeSH headings (or other standardized indexing terms) were used. The search was restricted to humans, but unrestricted for publication date or language (see supporting Table S1 for Medline search strategy. The search strategies for Embase and ISI were similar). We restricted the review to healthy people in order to separate potential associations of the SNPs with diabetes II, hypertension or with other diseases and only assess potential associations with overweight. This strategy was also followed by the previous review on the same topic by Paracchini and colleagues [15]. The reference lists of all included studies were examined to identify studies not found by the electronic databases search. The retrieved references were checked by title and abstract for inclusion or exclusion, according to the following criteria. Inclusion criteria: exposure: at least one of the *LEPR* SNPs Q223R, K109R or K656N; outcome: BMI, body fat percentage, weight, waist circumference, hip circumference, waist-to-hip ratio or other weight-related outcome; non related subjects, either sex, any ethnic group, any BMIs and all population based study designs. Exclusion criteria: studies not meeting the inclusion criteria; furthermore, studies including only non-healthy subjects (both study arms with e.g. diabetes, cancer or hypertension patients) and family-based studies. All included studies were retrieved as full text and reviewed again for inclusion and exclusion. The inclusion and exclusion process was performed at each level according to pre-established criteria by two independent reviewers (NB and NA), and consensus was reached by discussion.

Data was extracted from all the studies included as full text papers by the two independent reviewers on a standard data extraction sheet and entered into an electronic database (EpiData 3.1). Consensus was reached by discussion. Data extracted included reference details (author, year, journal), study design (case-control, cohort, comparative study), details of the population (sex, ethnicity, setting), sample size per comparison group, SNP studied, allele and genotype frequency per comparison group, obesity-related phenotypes tested (e.g. BMI, waist circumference), strength of association (odds ratios and confidence intervals), and potential confounders accounted for. If necessary data was not reported in the primary manuscript, the corresponding authors were contacted by email to request the missing data. Quality criteria were developed to assess the internal validity of the studies and the accuracy of reporting, using the guidelines for the assessment of cumulative evidence on genetic associations [20], the HuGe Review Handbook [21] and the extension of the STROBE statement STREGA (strengthening the reporting of genetic association studies) [22]. The quality of data was considered in the final interpretation of the findings.

### Data analysis and meta-analysis

Data was analysed descriptively and statistically for each *LEPR* gene variant separately and stratified by ethnic group. Ethnic groups were defined according to Rosenberg et al [23]. For the analysis of association between *LEPR* variants and overweight, case-control studies, (where cases are obese people without other known disease and controls are healthy non-obese people), were included. For genotype and allele frequencies, single group studies, (consisting of cohorts, cross-sectional studies or healthy control arms of case-control studies), were also analysed. Reported statistical analyses on the association between a SNP and an obesity-related outcome that did not present results in a format convenient for a meta-analysis, (such as linear regression, ANOVA, and non-parametric analyses), were extracted and taken into account in the interpretation of the findings.

Derived allele frequencies of each SNP were summarized in Tables, stratified by ethnic group (including CoLaus data, see below). If a study reported data separately for subgroups (such as sex, country or cohort of origin), the subgroups were included in the analysis of genotype and allele frequencies. The heterogeneity between allele frequencies in different ethnic groups was assessed using Cochran's Q statistic. In case-control studies, associations between genotype, allele data and obesity were assessed by chi-square tests, general linear models and meta-analyses, according to different inheritance models (co-dominant, dominant and recessive), given the *a priori* absence of evidence on the allelic mode of action. If odds ratios and/or confidence intervals were missing, these were calculated. We used 0.006 (0.05/9, because three models were tested for 3 SNPs) as the cut-off p-value to declare an association as significant in case-control studies (Tables 1–3).

**Table 1:** Analysis of genotypes for Q223R in case-control studies, according to different allelic modes of action.

Reference	Co-dominant: OR (95% CI)*	Co-dominant: p-value*	Dominant: OR (95% CI)**	Dominant: Chi^2^ (p-value)**	Recessive: OR (95% CI)**	Recessive: Chi^2^ (p-value)**
**Caucasians**						
Chagnon 1999 [61]	0.89 (0.74–1.07)	0.221	0.87 (0.52–1.45)	0.34 (0.557)	0.66 (0.36–1.19)	2.19 (0.139)
Yiannakouris 2001 [10]	1.45 (0.75–2.80)	0.263	0.96 (0.38–2.44)	0.01 (0.924)	5.54 (1.13–27.27)	7.40 (0.007)
Mattevi 2002 [31]	**1.62 (1.15–2.26)**	**0.005**	1.60 (0.99–2.59)	4.05 (0.044)	2.44 (1.16–5.12)	6.92 (0.009)
Portolés 2006 [62]	0.84 (0.68–1.04)	0104	0.88 (0.65–1.18)	0.84 (0.360)	0.62 (0.38–1.03)	3.96 (0.047)
De Krom 2007 [63]	1.00 (0.73–1.37)	0.991	0.77 (0.45–1.30)	1.11 (0.293)	1.33 (0.76–2.30)	1.14 (0.285)
Mergen 2007 [32]	1.43 (1.03–1.99)	0.034	1.65 (1.06–2.56)	5.45 (0.020)	1.31 (0.60–2.83)	0.55 (0.457)
Bienertova 2008 [64]	0.88 (0.57–1.36)	0.564	1.06 (0.50–2.23)	0.03 (0.872)	0.65 (0.30–1.44)	1.33 (0.248)
Masuo 2008 [16]	1.87 (1.10–3.16)	0.021	2.10 (0.90–4.90)	3.55 (0.060)	2.89 (0.88–9.49)	4.22 (0.040)
*Overall*	*I^2^ = 69.8%*	*I^2^ p = 0.002*	*1.13 (0.87–1.45)*	*0.368*	*I^2^ = 66.5%*	*I^2^ p = 0.004*
**Asians**						
Endo 2000 [65]	0.89 (0.58–1.37)	0.604	1.27 (0.20–8.25)	0.10 (0.754)	0.84 (0.49–1.42)	0.51 (0.475)
Wang 2006 [30]	1.32 (0.7–2.38)	0.347	1.51 (0.75–3.04)	1.57 (0.210)	0.39 (0.01–13.37)	0.61 (0.433)
*Overall*	*1.02 (0.71–1.47)*	*0.924*	*1.48 (0.77–2.85)*	*0.244*	*0.82 (0.49–1.38)*	*0.459*
**Mixed populations**						
Guízar-mendoza 2005 [66]	0.70 (0.36–1.34)	0.278	0.78 (0.33–1.84)	0.40 (0.527)	0.33 (0.04–2.74)	1.86 (0.173)
Duarte 2007 [67]	1.24 (0.89–1.71)	0.202	1.65 (1.02–2.68)	4.69 (0.030)	0.86 (0.45–1.65)	0.24 (0.628)
*Overall*	*1.00 (0.58–1.72)*	*0.991*	*1.24 (0.60–2.54)*	*0.564*	*0.79 (0.43–1.48)*	*0.465*
*Overall all populations*	*I^2^ = 56.7%*	*I^2^ p = 0.006*	*1.15 (0.93–1.43)*	*0.185*	*I^2^ = 52.0%*	*I^2^ p = 0.018*

Odds ratios and 95% confidence intervals are given. Statistically significant results are shown in bold. Where the overall measure was significantly heterogeneous, the I^2^ value and its p-value are given instead of the overall measure.

*Values from generalized linear model, overall results from meta-analysis, random model.

**Values from meta-analysis, random model.

**Table 2 pone-0026157-t002:** Analysis of genotypes for K109R in case-control studies, according to different allelic modes of action.

Reference	Co-dominant: OR (95% CI)*	Co-dominant: p-value*	Dominant: OR (95% CI)**	Dominant: Chi2 (p-value)**	Recessive: OR (95% CI)**	Recessive: Chi2 (p-value)**
**Caucasians**						
Chagnon 1999 [61]	0.82 (0.58–1.16)	0.253	0.94 (0.59–1.51)	0.07 (0.797)	0.38 (0.14–1.04)	4.70 (0.030)
Yiannakouris 2001 [10]	1.49 (0.62–3.61)	0.376	1.31 (0.45–3.83)	0.32 (0.574)	Not calculable	3.10 (0.079)
De Krom 2007 [63]	1.13 (0.78–1.62)	0.520	1.30 (0.77–2.18)	1.10 (0.294)	0.87 (0.34–2.23)	0.11 (0.739)
Masuo 2008 [16]	0.74 (0.37–1.48)	0.391	0.81 (0.34–1.93)	0.28 (0.599)	0.22 (0.01–7.46)	1.83 (0.177)
CoLaus men	0.91 (0.77–1.08)	0.298	0.91 (0.73–1.14)	0.67 (0.413)	0.81 (0.51–1.27)	0.95 (0.329)
CoLaus women	1.01 (0.86–1.18)	0.918	1.01 (0.82–1.23)	0.00 (0.959)	1.04 (0.67–1.60)	0.03 (0.872)
*Overall*	*0.96 (0.87–1.07)*	*0.449*	*0.98 (0.86–1.12)*	*0.782*	*0.84 (0.63–1.12)*	*0.242*
**Asians**						
Qu 2007 [68]	1.04 (0.77–1.41)	0.800	0.97 (0.34–2.76)	0.00 (0.950)	1.06 (0.73–1.53)	0.10 (0.750)
*Overall all populations*	*0.97 (0.88–1.07)*	*0.526*	*0.98 (0.86–1.12)*	*0.779*	*0.90 (0.72–1.13)*	*0.372*

Odds ratios and 95% confidence intervals are given. Statistically significant results are shown in bold.

*Values from generalized linear model, overall results from meta-analysis, random model.

**Values from meta-analysis, random model.

**Table 3:** Analysis of genotypes for K656N in case-control studies, according to different allelic modes of action.

Reference	Co-dominant: OR (95% CI)*	Co-dominant: p-value*	Dominant: OR (95% CI)**	Dominant: Chi2 (p-value)**	Recessive: OR (95% CI)**	Recessive: Chi2 (p-value)**
**Caucasians**						
Chagnon 1999 [61]	1.03 (0.72–1.48)	0.879	1.08 (0.66–1.77)	010 (0.752)	0.90 (0.33–2.46)	0.05 (0.820)
Yiannakouris 2001 [10]	1.15 (0.60–2.24)	0.670	1.25 (0.49–3.19)	0.27 (0.600)	1.03 (0.13–8.22)	0.00 (0.977)
Masuo 2008 [16]	1.71 (0.99–2.97)	0.055	1.97 (0.86–4.50)	3.12 (0.077)	2.28 (0.60–8.72)	2.04 (0.153)
*Overall*	*1.21 (0.89–1.63)*	*0.226*	*1.26 (0.86–1.86)*	*0.238*	*1.23 (0.58–2.59)*	*0.596*
**Asians**						
Qu 2007 [68]	0.87 (0.52–1.46)	0.587	0.91 (0.51–1.62)	0.12 (0.731)	Not calculable	1.42 (0.234)
*Overall all populations*	*1.12 (0.86–1.45)*	*0.407*	*1.14 (0.83–1.57)*	*0.421*		

Odds ratios and 95% confidence intervals are given. Statistically significant results are shown in bold.

*Results are from generalized linear model, overall results from meta-analysis, random model.

**Results are from meta-analysis, random model.

For the meta-analyses, data was pooled using a random effects model, to calculate summary odds ratios with 95% confidence intervals, by SNP. The statistical evidence for heterogeneity between studies was assessed by I^2^ statistics [24]. Funnel plots of study precision were used to examine a possible small study bias, using Begg and Egger statistics [25].

### CoLaus data

The CoLaus study (Cohorte Lausannoise) is a population-based study including 6'184 Caucasian adults aged 35–75 years from the city of Lausanne, Switzerland [17]. The study population consisted of 52.5% women. The mean age was 51.1 years (standard deviation of ±10.9). The following obesity-related phenotypic measurements were performed by trained nurses: body weight, body height, body fat percentage (by electrical bioimpedance using the Bodystat® 1500 analyzer [Isle of Man, British Isles]), waist and hip circumferences. Body weight and height were measured with participants standing without shoes in light indoor clothing. Body weight was measured in kilograms to the nearest 0.1 kg using a Seca® Scale (Hamburg, Germany), which was calibrated regularly. Height was measured to the nearest 5 mm using a Seca® height gauge (Hamburg, Germany). Waist circumference was measured twice with a nonstretchable tape over the unclothed abdomen at the mid-point between the lowest rib and the iliac crest. The mean of the two measurements was used for analyses. Furthermore, a number of additional potential confounders and effect modifiers were assessed by questionnaire or interview, including geographic origins [26], smoking status, alcohol consumption and menopausal status. Genotyping was performed using the Affymetrix 500K chip, 38 SNPs were located within the *LEPR* gene. In order to reduce the number of statistical tests performed, we chose 15 SNPs that tagged [27] all 38 SNPs (see complete list of covered SNPs on supporting Table S2), using the Haploview programme [28]. The 15 selected genotyped SNPs available at the *LEPR* locus tag 13, 26 and 33 SNPs from HapMap CEU release 22 using r^2^>0.8, >0.7 and >0.6, respectively, out of 208 SNPs (among which 188 SNPs have MAF >5%) available in this region. These tagging SNPs cover the entire gene region, including the 3′ UTR. We therefore consider that the set of SNPs we analyzed covers moderately well this locus. Relevant data on 5636 people was available for analysis in the present paper. Missing data was mostly due to missing genotype data.

We used logistic regressions to test the association of 15 tag *LEPR* SNPs (among them K109R) with the dichotomized body mass index (BMI) or waist circumference. Cut-off value for BMI was 25; cut-off value for waist circumference was 88 cm in women and 102 cm in men [29]. In addition, we performed linear regressions on body mass index, waist circumference, fat mass and leptin levels as continuous variables. Leptin level data was subjected to natural logarithmic transformation in order to better achieve normality of the residuals and homoscedasticity. We reported associations corrected for potential confounders like sex, age, height (for outcomes other than body mass index), alcohol consumption, smoking and geographic variation (expressed as principal components pc1 and pc2 from principal component analyses). These covariables showed an association with overweight-related phenotypes and with some of the SNPs studied in univariate regression analyses. We also assessed a potential interaction with sex. For analyses on the CoLaus data, we used 0.0033 (0.05/15) as the cut-off p-value to declare an association as significant. For interaction tests, we used 0.05 as the cut-off p-value to declare an interaction as significant. In the CoLaus analyses, we had more than 80% power to detect an additive association explaining 0.3% of trait variance for single SNP analysis and 80% power to detect an interaction explaining 0.14% of the variance.

All statistical analyses were conducted using STATA statistical package v 9.0 (Stata corp, College Station, TX, USA).

## Results

### Systematic review

**Figure 1:**
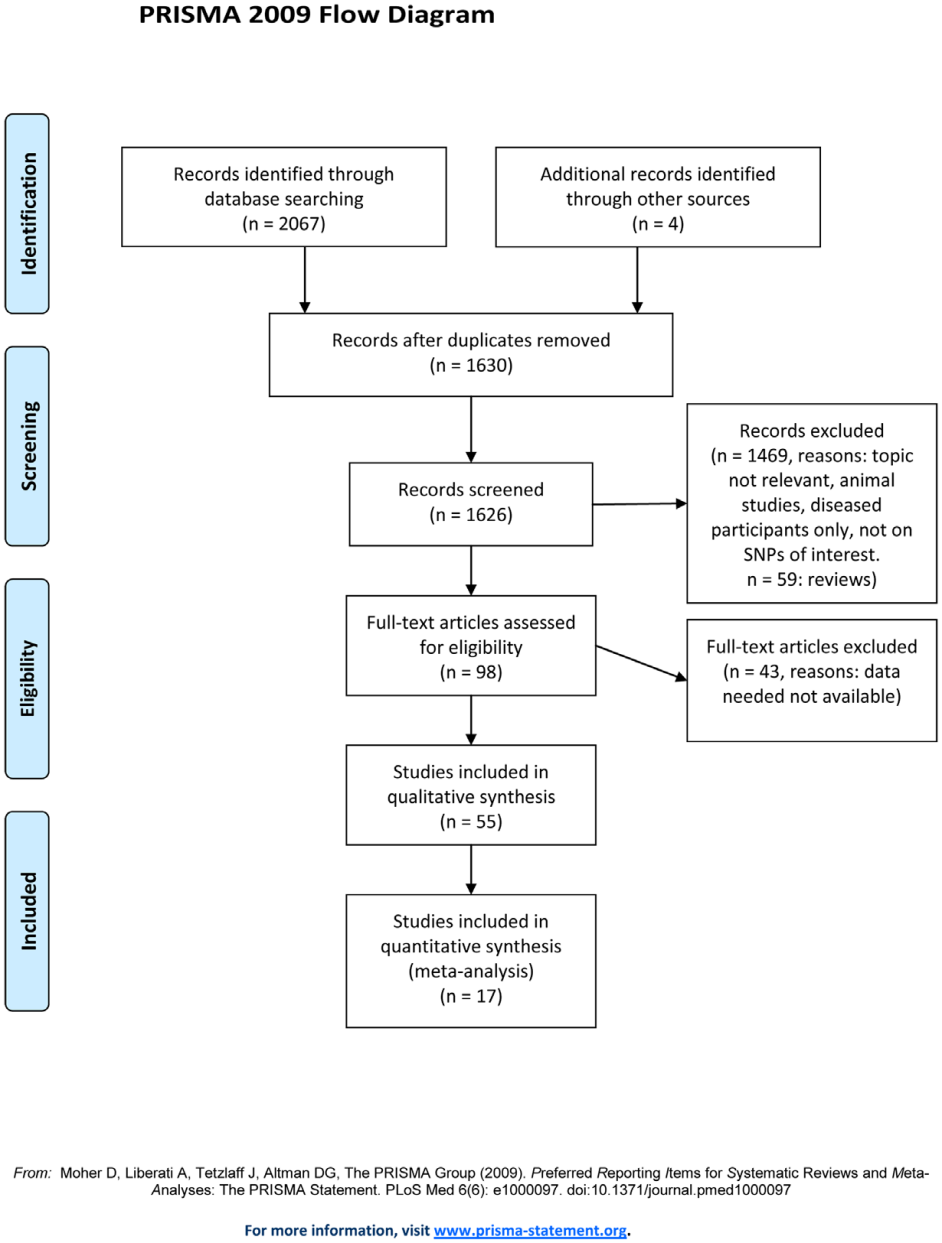
Flow diagram of studies included and excluded in the systematic review. Numbers are given at each exclusion step.

In total, 1630 papers were found through the search in electronic databases or by manual search. Fifty-five studies satisfied the inclusion and exclusion criteria and were obtainable as full text papers. Seventeen were case-control studies comparing obese with non-obese people and could be used for the meta-analyses. Thirty-eight studies were categorized as single group studies and contained data on genotype and allele frequencies (see the flow chart in Figure 1). supporting Table S3 presents participants' characteristics for case- control studies and supporting Table S4 for single group studies.

Thirty studies were carried out on Caucasians, sixteen studies were carried out on Asians, four on people of African ancestry and six on populations of mixed ancestry, such as Brazilians or Mexicans. Most studies reported BMI as outcome, many studies reported several obesity-related outcomes, like body weight, body fat mass or waist-to-hip ratio. Most studies were carried out with participants of both sexes and with adults. Q223R was the most commonly studied SNP (52 studies), followed by K109R (20 studies) and K656N (20 studies).

### Study quality

**Figure 2:**
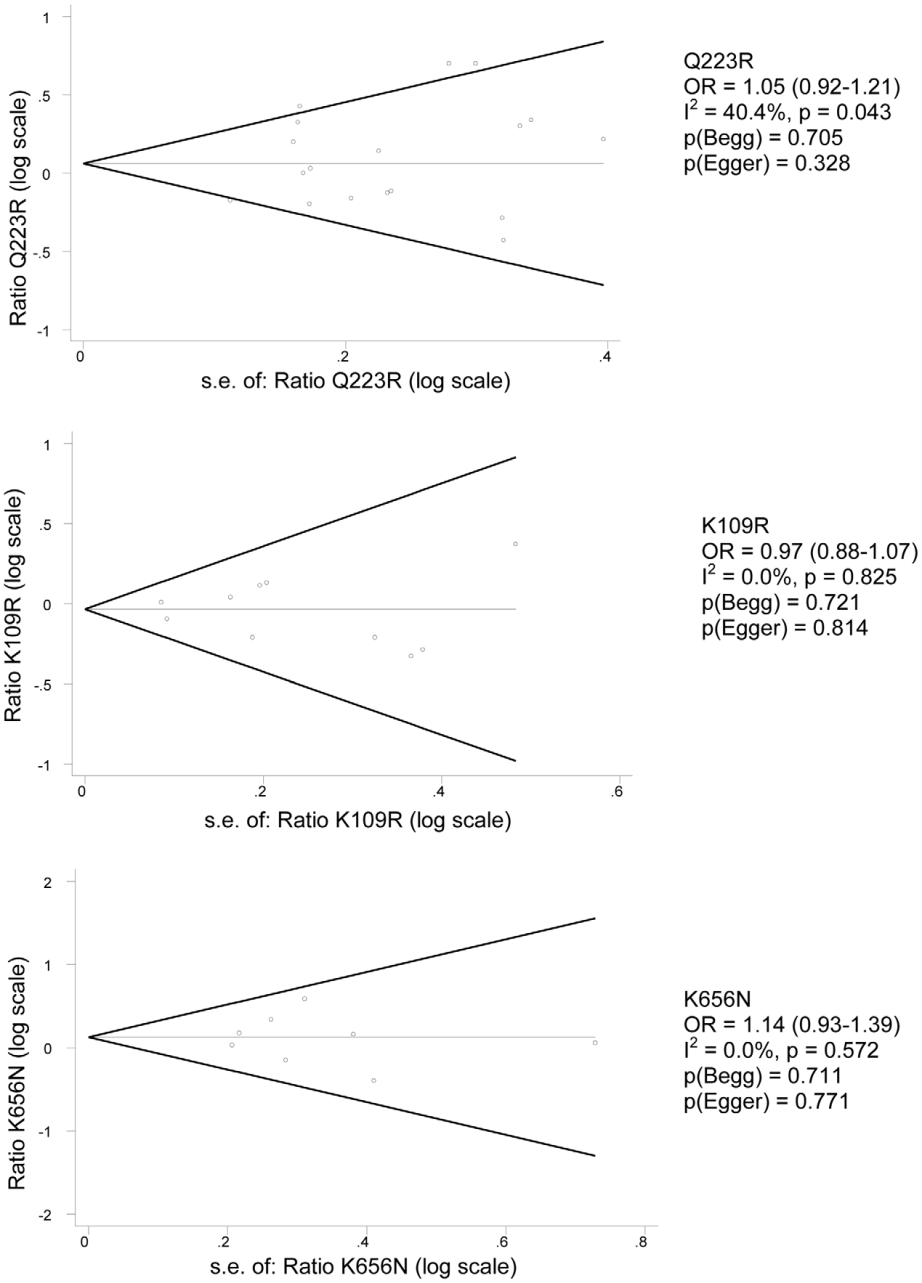
Begg's funnel plots of study precision. For included studies on the association between Q223R, K109R and K656N and overweight, Begg's funnel plots were used to examine a possible small study bias. The natural logarithm of the odds ratio (OR) vs. its standard error and pseudo 95% confidence intervals are shown, together with Begg and Egger statistics.

Overall, studies reported well on the participants' characteristics (summarized in supporting Tables S3 and S4) and on genotyping and analysis methods. Most studies did not report on missing data, and several call rates (the percentage of successfully genotyped individuals in the study population) were under 95%. Inclusion and exclusion criteria, as well as outcome assessments, were not always well described. Hardy-Weinberg equilibrium was mostly (but not always) reported as calculated. Genotypes were mostly in Hardy-Weinberg equilibrium. Potential confounders like sex and age were assessed in more than half of the studies. Surprisingly, the calculation or justification of the sample size was rarely described. The quality of study reporting is therefore of concern for the interpretation of the results. We assessed effects of small study bias by funnel plots (see Figure 2). There was no statistical evidence for publication bias or small study bias.

### Genotype and allele frequencies

**Figure 3:**
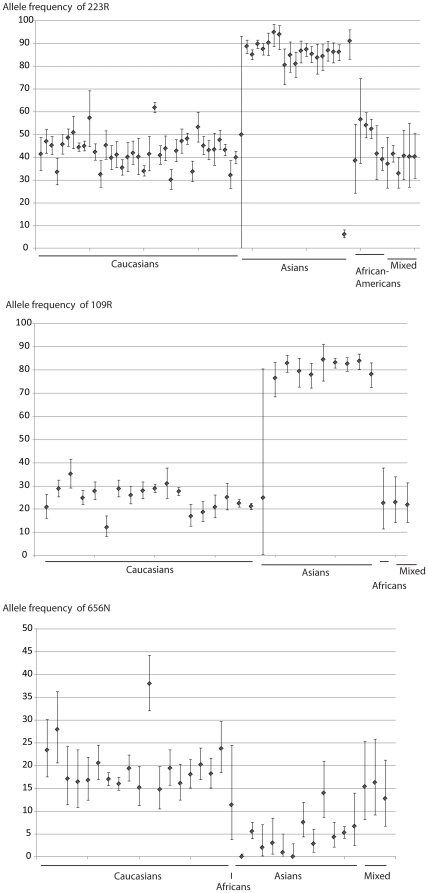
Derived allele frequencies for Q223R, K109R and K656N, by ethnic group. 95% confidence intervals are shown for each derived frequency.

In this systematic review, A denotes the ancestral allele and D the derived allele (the derived alleles are 223R, 109R and 656N respectively). Reported genotype and reported or calculated allele D frequencies with according 95% confidence intervals of all included studies are reported in Figure 3 and supporting Tables S5, S6, S7, by SNP. For each ethnic group the Cochran's Q statistic and p-value are given to estimate heterogeneity in allele frequencies across studies. Allele frequencies differ strongly between Caucasians and Asians, with Asians showing much higher derived allele frequencies for 223R (80.56–95.00% compared to 30.18–56.67% in Caucasians) and 109R (76.47–84.44 compared to 12.29–35.25 in Caucasians). The Taiwanese aborigines [30] have lower allele frequency (6.07%) for 223R, than the other Asian populations. Asians have lower 656N allele frequencies than Caucasians (0.00–13.97 vs. 14.75–37.98, respectively).

In Caucasians, allele frequencies are heterogeneous in all SNPs considered. For Q223R, there seems to be a north-south gradient in Caucasians, with the highest derived frequencies occurring in north Europe and the lowest derived frequencies found in Mediterranean countries.

### Meta-analyses and further results

**Figure 4:**
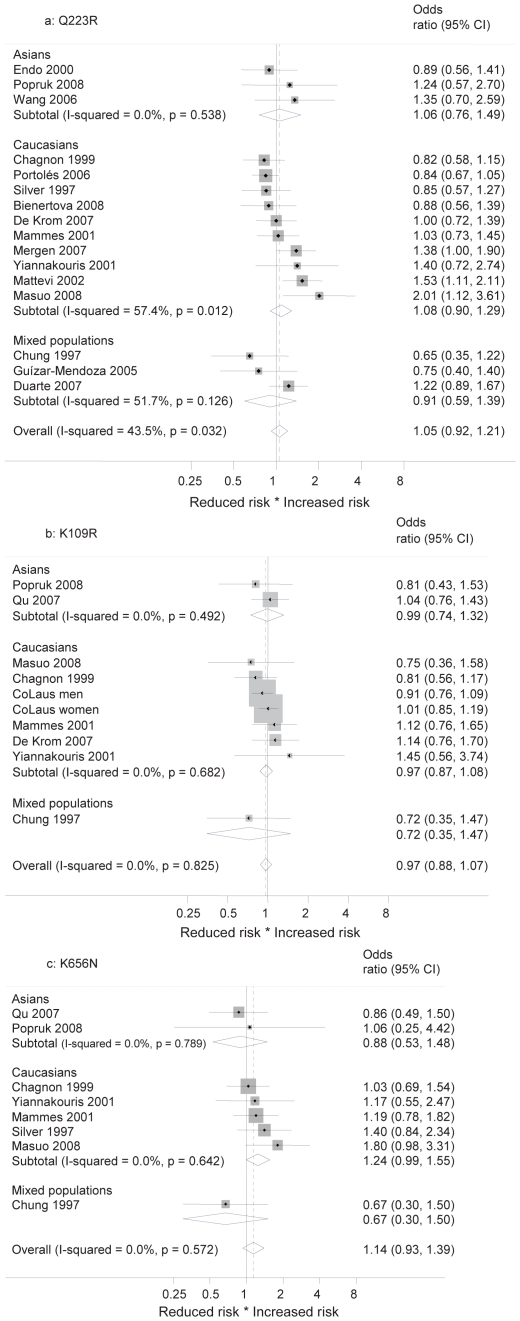
Forest plot on the association between SNP alleles and overweight in case-control studies by ethnicity. Figure 4a: Forest plot on the association between Q223R alleles and overweight in case-control studies by ethnicity. Overall association from random effects meta-analysis (odds ratio and 95% confidence intervals) and stratification by ethnic groups are shown, as well as heterogeneity by means of I^2^ value for overall measure and for subgroups. Figure 4b: Forest plot on the association between K109R alleles and overweight in case-control studies by ethnicity. Overall association from random effects meta-analysis (odds ratio and 95% confidence intervals) and stratification by ethnic groups are shown, as well as heterogeneity by means of I^2^ value for overall measure and for subgroups. Data from the CoLaus study are included for the Caucasian population, stratified by sex. Figure 4c: Forest plot on the association between K656N alleles and overweight in case-control studies by ethnicity. Overall association from random effects meta-analysis (odds ratio and 95% confidence intervals) and stratification by ethnic groups are shown, as well as heterogeneity by means of I^2^ value for overall measure and for subgroups.

Odds ratios of allele frequencies of case-control studies were analyzed by random effects meta-analyses. Results by SNPs are shown in Figures 4a–4c. No meta-analysis showed overall significant results (overall OR for Q223R: 1.05 (95% CI 0.92–1.21), for K109R: 0.97 (0.88–1.07), for K656N: 1.14 (0.93–1.39)). Pooled results from meta-analyses of genotypes showed no significant results in any SNP for any of the three inheritance models (see Tables 1–3).

**Figure 5:**
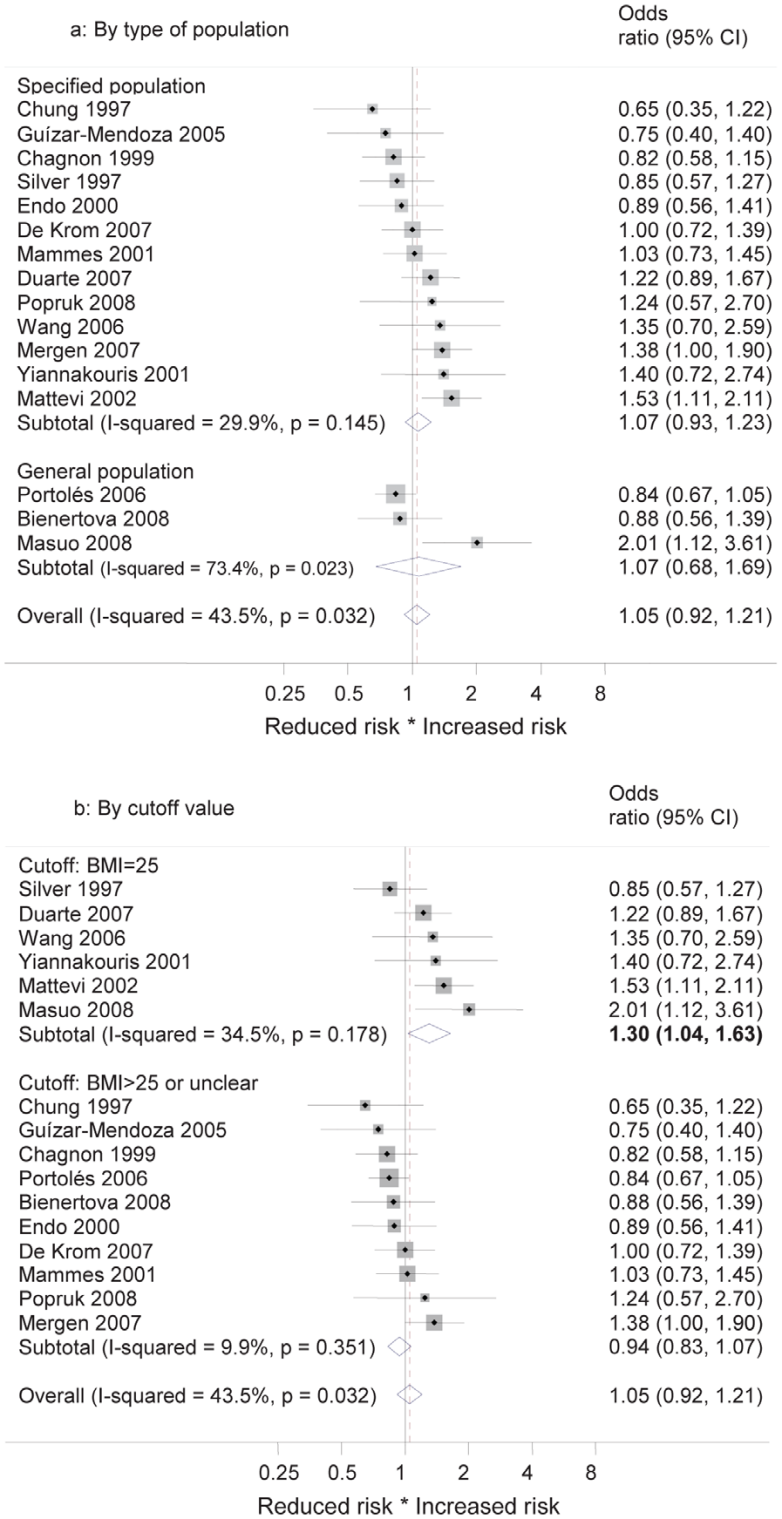
Forest plot on the association between Q223R alleles and overweight in case-control studies by potential effect modifier. Figure 5a: Forest plot on the association between Q223R alleles and overweight in case-control studies by type of population. Overall association from random effects meta-analysis (odds ratio and 95% confidence intervals) and stratification by type of population are shown, as well as heterogeneity by means of I^2^ value for overall measure and for subgroups. Specified populations are all populations not declared as general populations in the original studies. Figure 5b: Forest plot on the association between Q223R alleles and overweight in case-control studies by BMI cut-off value. Overall association from random effects meta-analysis (odds ratio and 95% confidence intervals) and stratification by BMI cut-off value are shown, as well as heterogeneity by means of I^2^ value for overall measure and for subgroups. For definition of BMI cut-off values see main text.

The meta-analysis of allele frequencies in Q223R showed significant heterogeneity (I^2^ = 43.5%, p = 0.032). Subgroup analyses of Q223R stratifying by ethnicity did not explain this heterogeneity and did not change any result to a significant association (Caucasians: OR 1.08 (0.90–1.29), Asians: OR 1.06 (0.76–1.49), mixed populations: OR 0.91 (0.59–1.39)), (see Figure 4a). The same is true for stratification by type of study population (see Figure 5a). However, if the meta-analysis of Q223R was stratified by BMI cutoff value, the heterogeneity became non-significant in each group (see Figure 5b). The overall OR for studies with a BMI cutoff value of 25 was significant with 1.30 (1.04–1.63). In studies using higher or unclear cutoff values the OR showed an opposite trend of 0.94 (0.83–1.07). Meta-analyses in K109R and K656N did not provide evidence for overall heterogeneity.

From the single studies on Q223R, only three studies showed an association between Q223R and obesity [16], [31], [32], reporting an increased risk of overweight for the derived allele (OR 2.1 (1.12–3.61), 1.53 (1.11–2.11), and 1.38 (1.00–1.90) respectively). One study reported data on the association between Q223R alleles and waist circumference stratified by sex [31]: in men the OR was 2.1 (1.16–3.47), showing an increased risk of large waist circumference for the derived allele. In women the OR was 1.15 (0.74–1.79). In our meta-analysis of genotypes in case-control studies there was only one single statistically significant result for Q223R genotypes with overweight in the co-dominant model (OR 1.62 (1.15–2.26) [31]). In the reported results from ANOVAs or linear regressions for Q223R, eight studies reported an increased risk of overweight for the derived allele, five a protective effect and 18 studies did not show an association.

For K109R, one study reported an association with overweight in women (p = 0.011) [33]. From ANOVAs or linear regressions, one study reported an increased risk for the derived allele, while nine studies did not show significant results.

In the results from ANOVAs or linear regressions for K656N, four studies reported an increased risk of the derived allele, while five studies did not show significant results.

### CoLaus data

Tables 4–5 show the results of the logistic regression analyses of 15 tag SNPs of the *LEPR* gene in CoLaus. For the association with overweight, only SNP rs9436746 showed an increased risk for the minor allele in the additive model (OR 1.13 (1.04–1.23)). No SNP showed an association with waist circumference and no SNP showed an interaction with sex. In the linear regressions (supporting Table S8), SNP rs10889553 showed an association with waist circumference in the additive model (beta 1.65 (SE 0.51), t-value 3.12, p = 0.001). In linear regressions three SNPs showed an interaction with sex (rs10128072, rs3790438 and rs3790437 (tag of K656N)) for the outcomes waist circumference and fat mass, and rs3790438 and rs3790437 showed an additional interaction with sex for the outcome BMI. If stratified by sex (supporting Table S9), all three SNPs show an increased risk for overweight-related outcomes for the minor alleles in men, and a decreased risk in women.

**Table 4:** Association from logistic regression models between *LEPR* variants and overweight in the CoLaus study.

SNP	Allele minor/major	OR (95% CI)heterozygote	OR (95% CI)homozygote minor	P(chi^2^, 2df)	OR (95% CI)additive	P add(1df)	P interaction SNP*sex
rs10128072	G/T	1.02 (0.9–1.16)	1.01 (0.71–1.44)	0.96	1.01 (0.91–1.13)	0.79	0.07
rs7518849	G/A	1.01 (0.85–1.20)	1.25 (0.43–3.69)	0.91	1.02 (0.87–1.20)	0.81	0.99
rs970467	T/C	0.91 (0.79–1.05)	0.72 (0.42–1.21)	0.21	0.90 (0.79–1.02)	0.09	0.58
rs10889553	T/C	1.14 (0.94–1.38)	2.72 (0.48–15.51)	0.21	1.16 (0.96–1.40)	0.12	0.21
rs10889567*	C/T	1.07 (0.94–1.22)	1.17 (0.99–1.38)	0.19	1.08 (0.99–1.17)	0.07	0.23
rs1137100**	G/A	1.05 (0.92–1.19)	1.14 (0.89–1.46)	0.51	1.06 (0.96–1.16)	0.26	0.86
rs3790438	A/T	0.87 (0.76–0.99)	0.94 (0.68–1.31)	0.10	0.90 (0.81–1.01)	0.07	0.08
**rs9436746**	A/C	1.11 (0.98–1.26)	1.29 (1.09–1.53)	0.01	**1.13 (1.04–1.23)**	**0.003**	0.25
rs2025805	A/G	0.82 (0.71–0.93)	0.87 (0.74–1.02)	0.01	0.93 (0.86–1.00)	0.06	0.35
rs1805096	T/C	1.01 (0.90–1.14)	0.88 (0.74–1.04)	0.22	0.95 (0.88–1.03)	0.25	0.51
rs9436748	T/G	0.84 (0.74–0.97)	0.94 (0.79–1.11)	0.04	0.95 (0.88–1.04)	0.25	0.14
rs7531110	G/T	1.04 (0.93–1.18)	1.18 (0.99–1.40)	0.18	1.08 (0.99–1.17)	0.08	0.16
rs10158279	C/A	1.01 (0.89–1.16)	1.13 (0.96–1.33)	0.25	1.06 (0.98–1.15)	0.15	0.41
rs11585329	A/C	1.05 (0.93–1.20)	0.88 (0.62–1.26)	0.53	1.02 (0.91–1.13)	0.78	0.57
rs3790437***	C/T	0.92 (0.81–1.04)	0.93 (0.70–1.25)	0.37	0.94 (0.85–1.03)	0.20	0.07

Results are odds ratios (95% confidence intervals) from logistic regression models (general model and additive model) including age, sex, alcohol consumption, smoking, and the first and second principal components, as covariates. In addition, the result of the interaction with sex is given. Statistically significant results are shown in bold.

*tag of Q223R.

**K109R.

***tag of K656N.

**Table 5:** Association from logistic regression models between *LEPR* variants and waist circumference in the CoLaus study.

SNP	Allele minor/major	OR (95% CI)heterozygote	OR (95% CI)homozygote minor	P(chi^2^, 2df)	OR (95% CI)additive	P add(1df)	P interaction SNP*sex
rs10128072	G/T	1.11 (0.97–1.28)	1.08 (0.74–1.58)	0.31	1.09 (0.97–1.23)	0.14	0.06
rs7518849	G/A	1.04 (0.87–1.25)	1.99 (0.70–5.66)	0.40	1.08 (0.91–1.29)	0.37	0.51
rs970467	T/C	0.98 (0.84–1.14)	0.53 (0.28–1.03)	0.14	0.93 (0.81–1.07)	0.32	0.56
rs10889553	T/C	1.19 (0.97–1.45)	1.94 (0.36–10.58)	0.18	1.20 (0.99–1.46)	0.06	0.08
rs10889567*	C/T	1.07 (0.93–1.23)	1.02 (0.85–1.22)	0.61	1.02 (0.93–1.11)	0.65	0.30
rs1137100**	G/A	1.01 (0.88–1.16)	0.93 (0.71–1.22)	0.85	0.99 (0.90–1.10)	0.91	0.25
rs3790438	A/T	0.90 (0.78–1.04)	0.90 (0.63–1.30)	0.32	0.92 (0.82–1.03)	0.16	0.16
rs9436746	A/C	1.05 (0.92–1.20	1.19 (0.99–1.42	0.17	1.09 (1.00–1.18)	0.06	0.29
rs2025805	A/G	0.87 (0.76–1.09)	0.92 (0.78–1.09)	0.18	0.95 (0.87–1.03)	0.22	0.22
rs1805096	T/C	1.02 (0.90–1.16)	0.84 (0.70–1.02)	0.12	0.95 (0.87–1.03)	0.21	0.53
rs9436748	T/G	0.85 (0.74–0.98)	0.95 (0.79–1.13)	0.07	0.95 (0.87–1.04)	0.24	0.34
rs7531110	G/T	1.00 (0.88–1.14)	1.03 (0.86–1.24)	0.94	1.02 (0.93–1.11)	0.71	0.06
rs10158279	C/A	1.05 (0.91–1.21)	1.04 (0.87–1.23)	0.82	1.02 (0.94–1.11)	0.63	0.17
rs11585329	A/C	1.06 (0.92–1.21)	0.96 (0.65–1.41)	0.68	1.03 (0.92–1.16)	0.59	0.58
rs3790437***	C/T	0.91 (0.80–1.04)	0.96 (0.70–1.31)	0.37	0.94 (0.84–1.05)	0.25	0.06

Results are odds ratios (95% confidence intervals) from logistic regression models (general model and additive model) including age, sex, height, alcohol consumption, smoking, and the first and second principal components, as covariates. In addition, the result of the interaction with sex is given. Statistically significant results are shown in bold.

*tag of Q223R.

**K109R.

***tag of K656N.

No SNP was associated in linear regressions with the outcome leptin levels. SNP rs7531110 showed an interaction with sex (supporting Tables S8 and S9).

## Discussion

### Overall results

In the present systematic review we analysed data on the association between three *LEPR* gene variants Q223R, K109R, K656N and overweight-related outcomes. In addition, we analysed primary data on the association of 15 *LEPR* tag SNPs with different overweight-related outcomes from a large, population based cross-sectional study. Overall, the meta-analysis of allele frequencies in obese cases and lean controls did not show an association between the three SNPs and overweight. In the present review, we also analysed genotype data, according to different genetic modes of action (co-dominant, dominant, recessive) and this did not reveal any clear pattern of association for any of the tested models. These results support previous findings [14], [15]. Studies published after our systematic review also confirm the unclear association between *LEPR* and overweight-related outcomes. Several studies reported no association between Q223R and overweight [34]–[36], while one study reported a protective effect of 223R for overweight in Pacific Islanders [37]. One study reported an increased risk of 109R for overweight in Asian children [38]. None of these studies would change our overall results. Most published studies are underpowered to detect small effect sizes. However, even in the large CoLaus study, we did not find evidence for an overall association between *LEPR* SNPs and overweight-related outcomes. Our search strategy is likely to have missed papers that included results on the association between the selected *LEPR* variants and obesity but have not mentioned *LEPR* in title or abstract. As these studies are likely to be negative, they would probably not change our overall conclusions.

### Implications of findings on interactions and non-coding variants

Stratification by factors that were reported in the literature as significant effect modifiers, like ethnicity [39] or study population [40] (general population versus specified populations) did not change the results. Interestingly, a stratification by BMI cut-off value [41] reduced the heterogeneity for Q223R within each of the two subgroups to non-significant levels. In the stratum with a cut-off value of BMI = 25, the result showed an increased risk for overweight for the derived allele. Okorodudu and colleagues [41] showed that a cutoff value of BMI = 25 had a sensitivity of 0.50 (CI: 0.43–0.57) and a specificity of 0.90 (0.86–0.94) in their study sample to detect high adiposity. A cutoff value of BMI = 30 had a sensitivity of 0.42 (0.31–0.43) and a specificity of 0.97 (0.96–0.97).

The fact that stratification by ethnicity did not explain overall heterogeneity and did not show a significant difference in the association between SNPs and overweight in the different ethnic groups is surprising, especially if one considers the allele frequency differences across ethnic groups. The present review supports previous findings of much higher derived allele frequencies in Asians for the SNPs Q223R and K109R [15], [42]. These higher derived allele frequencies are compatible with evidence for a recent positive natural selection of *LEPR* in Asian populations [43]. Interestingly, the Taiwanese aborigine population [30] shows a different picture. The derived allele of Q223R does not only show a lower frequency like in the Caucasian population, but even an extremely low frequency, compatible with a selection of the ancestral allele. This result could be explained by the fact that Taiwanese aborigines have a different evolutionary history compared to the other Asian populations included in the present review (Taiwanese aborigines separated before the selection of the *LEPR* variants in continental Asia occurred) [44]–[46].

Considering major sex differences in leptin levels and fat distribution, a further potential effect modifier is sex [47], [48]. We could not perform stratification by sex in our meta-analysis as most studies did not report associations separately in men and women. One study reported stratified allelic results for the outcome waist circumference [31], showing an increased risk for large waist circumference for 223R in men but not in women. Another study reported an association of K109R with overweight in women [33]. In our linear regressions of the CoLaus data SNPs rs10128072, rs3790438 and rs3790437 (tag of K656N) showed an interaction with sex in their association with waist circumference and fat mass. Sex can therefore be considered as a potential effect modifier for associations between *LEPR* SNPs and overweight-related outcomes. Two studies on the association between Q223R and overweight published after our systematic review confirm this view. One study reports an increased risk for 223R for high BMI in Caucasian girls but not in boys [49]. The other study reports a protective effect of 223R in Caucasian men but not in women [50]. These results suggest that stratification by sex should be recommended for future association studies.The three SNPs of the CoLaus data showing a significant interaction with sex are all non-coding variants of the *LEPR* gene, as is often the case for genetic associations using high-throughput DNA chips. This may indicate that these variants tag functional variants located within coding regions or functional variants located within non-coding regions influencing gene expression or splicing sites (promoter, introns, etc). It is more and more recognized that non-coding variants may impact on disease [51]. A better knowledge of the exact mechanisms of gene regulation will be crucial to understand the general role of non-coding DNA in human phenotypic variation.

### Evolutionary considerations

In our allele frequency data of Q223R we observed a north-south gradient in European Caucasians, with higher derived allele frequencies in the north and lower frequencies in the south. The same phenomenon for SNPs of other genes was reported in the Framingham Heart Study [52] and in a Europe wide analysis [26]. This phenomenon can be explained by the first settlement of Europe in Neolithic times from south to north [53]. It can lead to population stratification, a well known problem in large-scale association studies which can lead to false positive associations [39]. Furthermore, population stratification is a major issue in the interpretation of data from populations of known mixed ancestry, like Brazilians, Mexicans and certain US populations. For this reason, we considered such mixed populations separately in our systematic review and meta-analysis.

An alternative explanation for variation in allele frequency to the ancient migration hypothesis is positive natural selection. In fact, evidence for recent positive selection of variants in polymorphic genes was found in the major ethnic groups worldwide [43]. It was found that in selected genetic regions there is a significant over-representation of genetic association with complex diseases, a fact demonstrating that the understanding of recent genetic positive selection is important to comprehend the evolution of human disease [54]. Interestingly, the three *LEPR* SNPs Q223R, K109R and K656N show signals for positive selection in the Asian population [43]. This finding is compatible with the much higher derived allele frequencies for Q223R and K109R and the much lower derived allele frequency for K656N in Asians in our systematic review. It seems that the sequence changes were of advantage (or disadvantage) for Asian populations 6'000–8'000 years ago, a time that corresponds to the introduction of agriculture in Asia [55], [56]. It was therefore speculated that the *LEPR* gene could be considered a “thrifty” gene, leading to an accumulation of fat tissue in times of plenty, providing a reserve for times of hunger [57], [58]. As an alternative explanation for the positive selection of *LEPR* in Asian populations [59], Hancock et al. found associations of several *LEPR* variants (among them K109R) with climate variables suggesting a role of climate adaptations in the biological processes underlying cold adaptation and overweight. They suggest that variants like K109R might be deleterious in hot equatorial climates and advantageous in colder climates.

The influence of positive selection and the selective pressures operating in the past are issues of major importance that need further investigation. The human fat distribution, especially the subcutaneous fat, is unique among primates and among most land mammals. While primates have on average 5% body fat, a normal weight human male has approximately 10–15% body fat and a normal weight human female 20–25% [60]. Humans are born with a substantial layer of subcutaneous fat, showing the independence of this feature from diet at least in the first phase of life. The human subcutaneous fat and the susceptibility to obesity can therefore not only be explained by a disbalance between energy intake and expenditure, but also demands an evolutionary explanation.

### Conclusions

In conclusion, our systematic review did not show an overall association between the *LEPR* SNPs Q223R, K109R and K656N and obesity-related outcomes, but Q223R showed a significant association with overweight in studies considering a BMI cut-off value of 25 to separate normal weight from overweight. In our analyses of primary data from the CoLaus study, rs9436746 was associated with overweight and rs10889553 with waist circumference. Our stratified analyses in CoLaus data suggest that sex could potentially modify the association of *LEPR* variants with obesity-related phenotypes, which is not surprising considering the major differences in both leptin levels and fat distribution between the sexes. Genetic association studies on obesity traits should consider sex as a potential effect modifier. Finally, the role of natural selection in allele frequency differences and the potential impact of selection on gene-phenotype associations also need further investigation.

## Supporting Information

Table S1 **Search strategy for Medline, via platform OVID.**
(DOC)

Table S2 **List of 38 SNPs within the LEPR gene covered by the Affymetrix 500K chip. The 15 tag SNPs are in bold.**
(DOC)

Table S3 **Characteristics of case-control studies.**
(DOC)

Table S4 **Characteristics of cohort or cross-sectional studies or control arms of case-control studies.**
(DOC)

Table S5 **Genotype and derived allele frequencies (D) for Q223R, by ethnic group.**
(DOC)

Table S6 **Genotype and derived allele frequencies (D) for K109R, by ethnic group.**
(DOC)

Table S7 **Genotype and derived allele frequencies (D) for K656N, by ethnic group.**
(DOC)

Table S8 **Association from linear regression models of** ***LEPR*** **variants with different outcomes.**
(DOC)

Table S9 **Association from linear regression models of** ***LEPR*** **variants with different outcomes showing a significant interaction with sex, stratified by sex.**
(DOC)
