# Supplementary material for: Association between Variants of the Leptin Receptor Gene (LEPR) and Overweight: A Systematic Review and an Analysis of the CoLaus Study
Source: PLoS One. 2011 Oct 18;6(10):e26157. doi: 10.1371/journal.pone.0026157 (PMC3196514; doi:10.1371/journal.pone.0026157)
Supplement: Table S1 — Search strategy for Medline, via platform OVID. (DOC) [file pone.0026157.s001.doc]

**Supporting Table S1:** search strategy for Medline, via platform OVID

1. LEPR.mp.

2. leptin receptor gene.mp.

3. exp Receptors, Leptin/

4. Q223R.mp.

5. K109R.mp.

6. K656N.mp.

7. 1 or 2 or 3 or 4 or 5 or 6

8. exp Obesity/

9. exp Body Mass Index/

10. BMI.mp.

11. weight.mp.

12. exp Body Weight/

13. exp Waist-Hip Ratio/ or exp Waist Circumference/

14. WHR.mp.

15. body fat.mp. or exp Adipose Tissue/

16. exp Adiposity/

17. exp Overweight/

18. fat mass.mp.

19. Quetelet index.mp.

20. 8 or 9 or 10 or 11 or 12 or 13 or 14 or 15 or 16 or 17 or 18 or 19

21. 7 and 20

22. limit 21 to humans
