# Supplementary material for: Association between Variants of the Leptin Receptor Gene (LEPR) and Overweight: A Systematic Review and an Analysis of the CoLaus Study
Source: PLoS One. 2011 Oct 18;6(10):e26157. doi: 10.1371/journal.pone.0026157 (PMC3196514; doi:10.1371/journal.pone.0026157)
Supplement: Table S2 — List of 38 SNPs within the LEPR gene covered by the Affymetrix 500K chip. The 15 tag SNPs are in bold. (DOC) [file pone.0026157.s002.doc]

**Supporting Table S2:** list of 38 SNPs within the LEPR gene covered by the Affymetrix 500K chip. The 15 tag SNPs are in bold.

| rs10789184  rs1475398  rs10493379  rs3790425  rs12405556  rs7524834  rs3790426  rs7513047  rs2376018  **rs10128072**  **rs7518849**  rs11579567  **rs970467**  **rs10889553**  rs17097182  **rs10889567** (tag of Q223R)  **rs1137100** (K109R)  rs1751492  rs11208674 | rs6697315  **rs3790437** (tag of K656N)  **rs3790438**  rs2025803  **rs9436746**  rs11208679  rs17412368  **rs2025805**  rs17127838  **rs1805096**  **rs9436748**  **rs7531110**  **rs10158279**  rs1171261  rs2025804  rs12025906  rs1782755  **rs11585329**  rs4655518 |
| --- | --- |
