# Supplementary material for: Association between Variants of the Leptin Receptor Gene (LEPR) and Overweight: A Systematic Review and an Analysis of the CoLaus Study
Source: PLoS One. 2011 Oct 18;6(10):e26157. doi: 10.1371/journal.pone.0026157 (PMC3196514; doi:10.1371/journal.pone.0026157)
Supplement: Table S3 — Characteristics of case-control studies. (DOC) [file pone.0026157.s003.doc]

**Supporting Table S3:** characteristics of case-control studies

| **Reference** | **Population** | **Country** | **Sex** | **Age** | **Nr cases** | **Nr controls** | **SNP** | **Outcomes** | **Cut-off for outcome BMI** |
| --- | --- | --- | --- | --- | --- | --- | --- | --- | --- |
| **Caucasians** | | | | | | | | | |
| Chagnon 1999 [1] | Quebec Family Study | Canada | Males and females | Adults | 141 | 167 | Q223R K109R K656N | BMI, body weight, fat mass, body fat percentage, skin fold thickness | BMI 27 |
| Mammes 2001 [2] | Stanislas Family Study | France | Males and females | Cases: mean 42.2 +/- 5.1, controls: mean 40.9 +/- 4.4 | 98 | 289 | Q223R K109R K656N | BMI, body weight, fat mass | BMI 27 |
| Yiannakouris 2001 [3] | University students | Greece | Males and females | Range 14-26 | 29 | 89 | Q223R K109R K656N | BMI, body fat percentage, skin fold thickness | BMI 25 |
| Portolés 2006 [4] | Cases: endocrinology unit attenders, controls: general population | Spain | Males and females | Range 18-70 | 303 | 606 | Q223R | BMI, body weight | Cases: BMI>=30, controls: BMI<30 |
| De Krom 2007 [5] | Study population from European Prospective  Study into Cancer and Nutrition (EPIC) | The Netherlands | Females | Cases: mean 57 +/- 6 and mean 58 +/- 7, controls: 57 +/- 6 | 135 | 287 | Q223R K109R | BMI | BMI 33 |
| Mergen 2007 [6] | Endocrinology outpatients | Turkey | Males and females | Unclear | 262 | 138 | Q223R | BMI | Cases: BMI>=25 |
| Bienertova 2008 [7] | General population | Czech Republic | Males and females | Cases: mean 18.6-68.9, controls: mean 18.6-67.8 | 125 | 60 | Q223R | BMI, body weight, fat mass, body fat percentage, waist circumference, hip circumference, waist-to-hip ratio, skin fold thickness | Cases: BMI >=30 |
| Masuo 2008 [8] | General population | Australia | Males | Range 23-59 | 89 | 40 | Q223R K109R K656N | BMI | BMI 25 |
| **Asians** | | | | | | | | | |
| Endo 2000 [9] | School children | Japan | Males and females | Range 9-15 | 90 | 463 | Q223R | Obesity index: (real weight - standard weight) / standard weight x 100 | cases: obesity index of more than +20% |
| Wang 2006 [10] | General population | Taiwan | Males and females | >18 | 226 | 182 | Q223R | BMI, body weight, body fat percentage, waist circumference waist-to-hip ratio | Cases: BMI>=27, controls: BMI<25 |
| Qu 2007 [11] | General population | China | Males and females | Cases: mean 47.8, SD 10.5, controls: mean 46.7, SD 11.3 | 248 | 351 | K109R K656N | BMI | BMI 25 |
| Popruk 2008 [12] | Adolescents presenting at clinic for check-ups | Thailand | Males and females | Range 5-19 | 68 | 60 | Q223R K109R K656N | BMI, body weight, waist circumference, hip circumference | Normal: 15-85 percentile, risk for overweight: 85-95 perc, overweight: >95 perc. |
| **African Americans** | | | | | | | | | |
| Considine 1996 [13] | Autopsy cases | USA | Males | Cases: mean 45.1 +/- 4.9, controls: mean 46 +/- 7.1 | 8 | 7 | Q223R | BMI | cases: BMI 36.9 +/-1.5, controls: BMI 23.3 +/- 0.9 |
| **Mixed populations** | | | | | | | | | |
| Chung 1997 [14] | people attending medical centers | USA | Males and females | Adults and children | 167 | 27 | Q223R K109R K656N | BMI | unclear, obese: mean BMI 42.0, control: mean BMI 23.2 |
| Mattevi 2002 [15] | People attending for routine blood test | Brazil | Males and Females | Mean 39.9 years | 183 | 153 | Q223R | BMI, body weight, waist circumference | BMI 25 |
| Guízar-mendoza 2005 [16] | Unclear | Mexico | Males and Females | Range 12-17 | 55 | 48 | Q223R | BMI, body weight, body fat percentage, waist-to-hip ratio, skin fold thickness | cases: BMI>=95 percentile, controls: BMI 5-85 percentile |
| Duarte 2007 [17] | Cases: hypertension clinic patients, controls: blood donors | Brazil | Males and females | Range 18-71 | 200 | 150 | Q223R | BMI | Cases: BMI>30, controls: BMI<=24 |

**References**

1. Chagnon YC, Chung WK, Perusse L, Chagnon M, Leibel RL et al. (1999) Linkages and associations between the leptin receptor (LEPR) gene and human body composition in the Quebec Family Study. International Journal of Obesity & Related Metabolic Disorders: Journal of the International Association for the Study of Obesity 23: 278-286.

2. Mammes O, Aubert R, Betoulle D, Pean F, Herbeth B et al. (2001) LEPR gene polymorphisms: associations with overweight, fat mass and response to diet in women. Eur J Clin Invest 31: 398-404.

3. Yiannakouris N, Yannakoulia M, Melistas L, Chan JL, Klimis-Zacas D et al. (2001) The Q223R polymorphism of the leptin receptor gene is significantly associated with obesity and predicts a small percentage of body weight and body composition variability. Journal of Clinical Endocrinology & Metabolism 86: 4434-4439.

4. Portoles O, Sorli JV, Frances F, Coltell O, Gonzalez JI et al. (2006) Effect of genetic variation in the leptin gene promoter and the leptin receptor gene on obesity risk in a population-based case-control study in Spain. Eur J Epidemiol 21: 605-612.

5. de Krom M, van der Schouw YT, Hendriks J, Ophoff RA, van Gils CH et al. (2007) Common genetic variations in CCK, leptin, and leptin receptor genes are associated with specific human eating patterns. Diabetes 56: 276-280.

6. Mergen H, Karaaslan C, Mergen M, Deniz OE, Ozata M (2007) LEPR, ADBR3, IRS-1 and 5-HTT genes polymorphisms do not associate with obesity. Endocr J 54: 89-94.

7. Bienertova-Vasku J, Bienert P, Tomandl J, Forejt M, Vavrina M et al. (2008) No association of defined variability in leptin, leptin receptor, adiponectin, proopiomelanocortin and ghrelin gene with food preferences in the Czech population. Nutr Neurosci 11: 2-8.

8. Masuo K, Straznicky NE, Lambert GW, Katsuya T, Sugimoto K et al. (2008) Leptin-receptor polymorphisms relate to obesity through blunted leptin-mediated sympathetic nerve activation in a Caucasian male population.[see comment]. Hypertension Research - Clinical & Experimental 31: 1093-1100.

9. Endo K, Yanagi H, Hirano C, Hamaguchi H, Tsuchiya S et al. (2000) Association of Trp64Arg polymorphism of the beta3-adrenergic receptor gene and no association of Gln223Arg polymorphism of the leptin receptor gene in Japanese schoolchildren with obesity. International Journal of Obesity & Related Metabolic Disorders: Journal of the International Association for the Study of Obesity 24: 443-449.

10. Wang TN, Huang MC, Chang WT, Ko AM, Tsai EM et al. (2006) G-2548A polymorphism of the leptin gene is correlated with extreme obesity in Taiwanese aborigines. Obesity 14: 183-187.

11. Qu Y, Yang Z, Jin F, Sun L, Zhang C et al. (2007) Analysis of the relationship between three coding polymorphisms in LEPR gene and obesity in northern Chinese. Obes Res Clin Pract 1: 261-266.

12. Popruk S, Tungtrongchitr R, Petmitr S, Pongpaew P, Harnroongroj T et al. (2008) Leptin, soluble leptin receptor, lipid profiles, and LEPR gene polymorphisms in Thai children and adolescents. International Journal for Vitamin & Nutrition Research 78: 9-15.

13. Considine RV, Considine EL, Williams CJ, Hyde TM, Caro JF (1996) The hypothalamic leptin receptor in humans: identification of incidental sequence polymorphisms and absence of the db/db mouse and fa/fa rat mutations. Diabetes 45: 992-994.

14. Chung WK, Power-Kehoe L, Chua M, Chu F, Aronne L et al. (1997) Exonic and intronic sequence variation in the human leptin receptor gene (LEPR). Diabetes 46: 1509-1511.

15. Mattevi VS, Zembrzuski VM, Hutz MH (2002) Association analysis of genes involved in the leptin-signaling pathway with obesity in Brazil. International Journal of Obesity & Related Metabolic Disorders: Journal of the International Association for the Study of Obesity 26: 1179-1185.

16. Guizar-Mendoza JM, Amador-Licona N, Flores-Martinez SE, Lopez-Cardona MG, Ahuatzin-Tremary R et al. (2005) Association analysis of the Gln223Arg polymorphism in the human leptin receptor gene, and traits related to obesity in Mexican adolescents.[see comment]. J Hum Hypertens 19: 341-346.

17. Duarte SF, Francischetti EA, Genelhu VA, Cabello PH, Pimentel MM (2007) Lepr p.Q223r, beta3-ar p.W64r and lep c.-2548G>A gene variants in obese brazilian subjects. Genetics & Molecular Research 6: 1035-1043.
