# Supplementary material for: Association between Variants of the Leptin Receptor Gene (LEPR) and Overweight: A Systematic Review and an Analysis of the CoLaus Study
Source: PLoS One. 2011 Oct 18;6(10):e26157. doi: 10.1371/journal.pone.0026157 (PMC3196514; doi:10.1371/journal.pone.0026157)
Supplement: Table S4 — Characteristics of cohort or cross-sectional studies or control arms of case-control studies. (DOC) [file pone.0026157.s004.doc]

**Supporting Table S4:** characteristics of cohort or cross-sectional studies or control arms of case-control studies

| **Reference** | **Population** | | **Country** | **Sex** | **Age** | **Nr of participants** | **SNP** | **Outcomes** |
| --- | --- | --- | --- | --- | --- | --- | --- | --- |
| **Caucasians** | | | | | | | | |
| Silver 1997 [1] | | Baltimore Longitudinal Study on Aging and obese people attending the Johns Hopkins University Weight Management Center | USA | Males and females | Cases: mean 61.5 +/- 13.5, controls: mean 56.7 +/- 18.3, cohort: mean 42.8 +/- 11.2 | 388 | Q223R K656N | BMI, cases: BMI>26.7, controls: BMI<23.3 |
| Oksanen 2000 [2] | | Blood donors | Finland | Females | Range 40-50 | 122 | Q223R K109R K656N | BMI |
| Quinton 2001 [3] | | Postmenopausal women | United Kingdom | Females | Unclear | 89 | Q223R | BMI, fat mass |
| Rand 2001 [4] | | Pregnant women | United Kingdom, India, Bangladesh, Pakistan | Females | Mean 31.63 sd 4.71 and 30.77, SD 4.35 | 455 | Q223R | BMI |
| Wauters 2001 [5] | | Obese outpatients | Belgium | Females | Range 18-60 | 280 | Q223R K109R K656N | BMI, body weight, fat mass, waist circumference, hip circumference |
| Van Rossum 2002 [6] | | Cohort studies on cardiovascular risk factors | The Netherlands | Males and females | Range 20-40 | 582 | Q223R K109R K656N | BMI, body weight |
| Skibola 2004 [7] | | General population | USA | Males and females | Range 21-74 | 805 | Q223R | BMI |
| Willett 2005 [8] | | General population | United Kingdom | Males and females | Range 18-64 | 754 | Q223R | BMI |
| Banerjee 2006 [9] | | Attenders of paediatric endocrine outpatient clinics | United Kingdom | Males and females | Range 8-18 | 94 | Q223R | Not relevant |
| Crabbe 2006 [10] | | General population | Belgium | Males | Range 71-86 | 270 | Q223R | BMI, fat mass |
| Snoussi 2006 [11] | | Controls: blood donors | Tunisia | Females | Range 34-62 | 222 | Q223R | Not relevant |
| Fairbrother 2007 [12] | | General population | Denmark | Females | Range 60-84 | 1306 | Q223R K109R | BMI, body weight, fat mass, body fat percentage, |
| Popko 2007 [13] | | Unclear | Poland | Males and females | Range 18-65 | 77 | Q223R | Not relevant |
| Richert 2007 [14] | | Attenders of public health youth setting | Switzerland | Males | Mean 7.4, SE 0.03 | 222 | Q223R | BMI, body weight |
| Zhang 2007 [15] | | Employees | Italy | Males and females | Males: mean 39.5, SD 8.2. females: mean 36.1, SD 8.3 | 630 | Q223R | BMI, waist circumference |
| Den Hoed 2008 [16] | | General population | The Netherlands | Males and females | Mean 31 +/- 14 years | 103 | Q223R K109R | BMI |
| De Luis 2008 [17] | | Obese attenders of outpatient nutrition clinic | Spain | Males and females | Range 27-60.2 | 231 | K656N | BMI, body weight, fat mass, waist circumference, hip circumference, waist-to-hip ratio |
| Doecke 2008 [18] | | General population | Australia | Males and females | Unclear | 1352 | Q223R K109R K656N | BMI |
| Iciek 2008 [19] | | Pregnant women | Poland | Females | Unclear | 34 | Q223R | BMI, body weight |
| Abete 2009[20] | | Unclear | Spain | Males and females | Range 20-50 | 170 | K109R | BMI, body weight, fat mass, waist circumference |
| Marti 2009 [21] | | Attenders of the Endocrinology and Occupational Health Department, Navarra Hosp. | Spain | Males and females | Mean 38.6 +/- 9.0 | 154 | Q223R K109R K656N | BMI, body weight, body fat percentage |
| Szczepankiewicz 2009 [22] | | General population | Poland | Males and females | Males: mean 10, SD 2.2, females: mean 9.6, SD 1.8 | 114 | Q223R K109R | BMI |
| Vasku 2009 [23] | | Unclear | Czech Republic | Males and females | Range 62.7-73.5 | 101 | Q223R | BMI |
| **Asians** | | | | | | | | |
| Matsuoka 1997 [24] | Unclear | | Japan | Males and females | Mean 45.9 +/- 8.0 | 68 | Q223R K109R K656N | BMI |
| De Silva 1999[25] | Other cohort study | | Nauru | Males | Mean 31 | 232 | Q223R | BMI |
| Koh 2002[26] | University students | | South Korea | Males | Range 20-34 | 219 | Q223R K109R | BMI, body weight |
| Huang 2003 [27] | Northern Han Chinese | | China | Males and females | Mean 43.92 +/- 1.16 | 78 | Q223R | BMI |
| Kagawa 2003 [28] | General population | | Japan, Palau, Thailand | Males and females | Between mean 58, SE 0.6 and 67, SE 1.0 | 430 | Q223R K656N | BMI |
| Takahashi Yasuno 2003 [29] | People not taking medical care | | Japan | Males | Range 36-66 | 201 | Q223R | BMI |
| Ogawa 2004 [30] | Healthy subjects receiving annual check up | | Japan | Males and females | Males: mean 44.5 +/- 9.3, females: mean 41.7 +/- 9.7 | Males: 127, females: 90 | Q223R K109R | BMI |
| Woo 2006 [31] | Women presenting at health screening center | | South Korea | Females | Unclear, pre- and post-menopausal | 45 | Q223R K109R K656N | BMI |
| Han C.Z. 2008 [32] | Hospital attenders | | China | Females | Range 22-78 | 500 | Q223R | BMI, body weight, waist circumference, hip circumference, waist-to-hip ratio |
| Han H.R. 2008 [33] | Hospital attenders | | South Korea | Females | Mean 64.47, SD 3.36 | 345 | Q223R K109R K656N | BMI |
| Kim 2008 [34] | Women attending routine health check | | South Korea | Females | 24-81 | 263 | Q223R K109R | BMI, body weight, fat mass |
| **Mixed populations** | | | | | | | | |
| Méndez-Sánchez 2006 [35] | Persons presenting for routine check-up | | Mexico | Males and females | Mean 44.83, SD 8.704 | 43 | Q223R K656N | BMI, body weight, body fat percentage, waist circumference, hip circumference, waist-to-hip ratio |
| Roth 2005 [36] | Women's Alcohol Study participants | | USA | Females | Postmenopausal | 53 | Q223R K109R K656N | Body weight |
| Podolsky 2007 [37] | Study population from longitudinal study of development of cardiovascular risk factors | | USA | Males and females | Range 3.9-23.9 | 526 | Q223R | BMI, body weight, waist circumference, skin fold thickness |
| **Black populations** | | | | | | | | |
| Ragin 2009 [38] | Study on cervical and oral HPV infection, study on polymorphisms and tobacco metabolism | | Caribbean Islands, USA, Africa | Females | Mean 41.1, 46.5, 44.7, 50.1 | 1418 | Q223R | BMI |

**References**

1. Silver K, Walston J, Chung WK, Yao F, Parikh VV et al. (1997) The Gln223Arg and Lys656Asn polymorphisms in the human leptin receptor do not associate with traits related to obesity. Diabetes 46: 1898-1900.

2. Oksanen L, Tiitinen A, Kaprio J, Koistinen HA, Karonen S et al. (2000) No evidence for mutations of the leptin or leptin receptor genes in women with polycystic ovary syndrome. Mol Hum Reprod 6: 873-876.

3. Quinton ND, Lee AJ, Ross RJ, Eastell R, Blakemore AI (2001) A single nucleotide polymorphism (SNP) in the leptin receptor is associated with BMI, fat mass and leptin levels in postmenopausal Caucasian women. Hum Genet 108: 233-236.

4. Rand L, Winchester EC, Millwood IY, Penny MA, Kessling AM (2001) Maternal leptin receptor gene variant Gln223Arg is not associated with variation in birth weight or maternal body mass index in UK and South Asian populations. International Journal of Obesity & Related Metabolic Disorders: Journal of the International Association for the Study of Obesity 25: 753-755.

5. Wauters M, Mertens I, Chagnon M, Rankinen T, Considine RV et al. (2001) Polymorphisms in the leptin receptor gene, body composition and fat distribution in overweight and obese women. International Journal of Obesity & Related Metabolic Disorders: Journal of the International Association for the Study of Obesity 25: 714-720.

6. van Rossum CT, Hoebee B, Seidell JC, Bouchard C, van Baak MA et al. (2002) Genetic factors as predictors of weight gain in young adult Dutch men and women. International Journal of Obesity & Related Metabolic Disorders: Journal of the International Association for the Study of Obesity 26: 517-528.

7. Skibola CF, Holly EA, Forrest MS, Hubbard A, Bracci PM et al. (2004) Body mass index, leptin and leptin receptor polymorphisms, and non-hodgkin lymphoma. Cancer Epidemiology, Biomarkers & Prevention 13: 779-786.

8. Willett EV, Skibola CF, Adamson P, Skibola DR, Morgan GJ et al. (2005) Non-Hodgkin's lymphoma, obesity and energy homeostasis polymorphisms. Br J Cancer 93: 811-816.

9. Banerjee I, Trueman JA, Hall CM, Price DA, Patel L et al. (2006) Phenotypic variation in constitutional delay of growth and puberty: relationship to specific leptin and leptin receptor gene polymorphisms. Eur J Endocrinol 155: 121-126.

10. Crabbe P, Goemaere S, Zmierczak H, Van P, I, De Bacquer D et al. (2006) Are serum leptin and the Gln223Arg polymorphism of the leptin receptor determinants of bone homeostasis in elderly men? Eur J Endocrinol 154: 707-714.

11. Snoussi K, Strosberg AD, Bouaouina N, Ben Ahmed S, Helal AN et al. (2006) Leptin and leptin receptor polymorphisms are associated with increased risk and poor prognosis of breast carcinoma. Bmc Cancer 6.

12. Fairbrother UL, Tanko LB, Walley AJ, Christiansen C, Froguel P et al. (2007) Leptin receptor genotype at Gln223Arg is associated with body composition, BMD, and vertebral fracture in postmenopausal Danish women. Journal of Bone & Mineral Research 22: 544-550.

13. Popko K, Gorska E, Wasik M, Stoklosa A, Plywaczewski R et al. (2007) Frequency of distribution of leptin receptor gene polymorphism in obstructive sleep apnea patients. Journal of Physiology & Pharmacology 58: 551-561.

14. Richert L, Chevalley T, Manen D, Bonjour JP, Rizzoli R et al. (2007) Bone mass in prepubertal boys is associated with a Gln223Arg amino acid substitution in the leptin receptor. Journal of Clinical Endocrinology & Metabolism 92: 4380-4386.

15. Zhang YY, Gottardo L, Mlynarski W, Frazier W, Nolan D et al. (2007) Genetic variability at the leptin receptor (LEPR) locus is a determinant of plasma fibrinogen and C-reactive protein levels. Atherosclerosis 191: 121-127.

16. den Hoed M, Smeets AJ, Veldhorst MA, Nieuwenhuizen AG, Bouwman FG et al. (2008) SNP analyses of postprandial responses in (an)orexigenic hormones and feelings of hunger reveal long-term physiological adaptations to facilitate homeostasis. Int J Obes (Lond) 32: 1790-1798.

17. de Luis DA, Gonzalez SM, Aller R, Izaola O, Conde R (2008) Influence of Lys656Asn polymorphism of the leptin receptor gene on insulin resistance in nondiabetic obese patients. Journal of Diabetes & its Complications 22: 199-204.

18. Doecke JD, Zhao ZZ, Stark MS, Green AC, Hayward NK et al. (2008) Single nucleotide polymorphisms in obesity-related genes and the risk of esophageal cancers. Cancer Epidemiology, Biomarkers & Prevention 17: 1007-1012.

19. Iciek R, Wender-Ozegowska E, Seremak-Mrozikiewicz A, Drews K, Sodowski K et al. (2008) Leptin gene, leptin gene polymorphisms and body weight in pregnant women with diabetes mellitus type I. Journal of Physiology & Pharmacology 59: Suppl-31.

20. Abete I, Goyenechea E, Crujeiras AB, Martinez JA (2009) Inflammatory State and Stress Condition in Weight-lowering Lys109Arg LEPR Gene Polymorphism Carriers. Arch Med Res 40: 306-310.

21. Marti A, Santos JL, Gratacos M, Moreno-Aliaga MJ, Maiz A et al. (2009) Association between leptin receptor (LEPR) and brain-derived neurotrophic factor (BDNF) gene variants and obesity: a case-control study. Nutr Neurosci 12: 183-188.

22. Szczepankiewicz A, Breborowicz A, Sobkowiak P, Popiel A (2009) Are genes associated with energy metabolism important in asthma and BMI? J Asthma 46: 53-58.

23. Vasku A, Vokurka J, Bienertova-Vasku J (2009) Obesity-related genes variability in Czech patients with sporadic colorectal cancer: preliminary results. Int J Colorectal Dis 24: 289-294.

24. Matsuoka N, Ogawa Y, Hosoda K, Matsuda J, Masuzaki H et al. (1997) Human leptin receptor gene in obese Japanese subjects: evidence against either obesity-causing mutations or association of sequence variants with obesity. Diabetologia 40: 1204-1210.

25. de Silva AM, Walder KR, Aitman TJ, Gotoda T, Goldstone AP et al. (1999) Combination of polymorphisms in OB-R and the OB gene associated with insulin resistance in Nauruan males. International Journal of Obesity & Related Metabolic Disorders: Journal of the International Association for the Study of Obesity 23: 816-822.

26. Koh JM, Kim DJ, Hong JS, Park JY, Lee KU et al. (2002) Estrogen receptor alpha gene polymorphisms Pvu II and Xba I influence association between leptin receptor gene polymorphism (Gln223Arg) and bone mineral density in young men. Eur J Endocrinol 147: 777-783.

27. Huang R, Huang XZ, Li M, Xiao Y, Zhang J (2003) [An investigation of the relationship between Lepr gene Gln223Arg polymorphism and obstructive sleep apnea hypopnea syndrome]. [Chinese]. Chung-Hua Chieh Ho Ho Hu Hsi Tsa Chih Chinese Journal of Tuberculosis & Respiratory Diseases 26: 517-521.

28. Kagawa Y, Dever GJ, Otto CT, Charupoonphol P, Supannatas S et al. (2003) Single nucleotide polymorphism and lifestyle-related diseases in the Asia-Pacific region: comparative study in Okinawa, Palau and Thailand. Asia Pac J Public Health 15 Suppl: S10-S14.

29. Takahashi-Yasuno A, Masuzaki H, Miyawaki T, Ogawa Y, Matsuoka N et al. (2003) Leptin receptor polymorphism is associated with serum lipid levels and impairment of cholesterol lowering effect by simvastatin in Japanese men. Diabetes Research & Clinical Practice 62: 169-175.

30. Ogawa T, Hirose H, Yamamoto Y, Nishikai K, Miyashita K et al. (2004) Relationships between serum soluble leptin receptor level and serum leptin and adiponectin levels, insulin resistance index, lipid profile, and leptin receptor gene polymorphisms in the Japanese population. Metabolism: Clinical & Experimental 53: 879-885.

31. Woo HY, Park H, Ki CS, Park YL, Bae WG (2006) Relationships among serum leptin, leptin receptor gene polymorphisms, and breast cancer in Korea. Cancer Lett 237: 137-142.

32. Han CZ, Du LL, Jing JX, Zhao XW, Tian FG et al. (2008) Associations among lipids, leptin, and leptin receptor gene Gin223Arg polymorphisms and breast cancer in China. Biol Trace Elem Res 126: 38-48.

33. Han HR, Ryu HJ, Cha HS, Go MJ, Ahn Y et al. (2008) Genetic variations in the leptin and leptin receptor genes are associated with type 2 diabetes mellitus and metabolic traits in the Korean female population. Clin Genet 74: 105-115.

34. Kim SM, Kim SH, Lee JR, Jee BC, Ku SY et al. (2008) Association of leptin receptor polymorphisms Lys109Arg and Gln223Arg with serum leptin profile and bone mineral density in Korean women. Am J Obstet Gynecol 198: 421-428.

35. Mendez-Sanchez N, Bermejo-Martinez L, Chavez-Tapia NC, Zamora-Valdes D, Sanchez-Lara K et al. (2006) Obesity-related leptin receptor polymorphisms and gallstones disease. Ann Hepatol 5: 97-102.

36. Roth MJ, Paltoo DN, Albert PS, Baer DJ, Judd JT et al. (2005) Common leptin receptor polymorphisms do not modify the effect of alcohol ingestion on serum leptin levels in a controlled feeding and alcohol ingestion study. Cancer Epidemiology, Biomarkers & Prevention 14: 1576-1578.

37. Podolsky RH, Barbeau P, Kang HS, Zhu H, Treiber FA et al. (2007) Candidate genes and growth curves for adiposity in African- and European-American youth. Int J Obes 31: 1491-1499.

38. Ragin CC, Dallal C, Okobia M, Modugno F, Chen J et al. (2009) Leptin levels and leptin receptor polymorphism frequency in healthy populations. Infect Agents Cancer 4.
