# Supplementary material for: Association between Variants of the Leptin Receptor Gene (LEPR) and Overweight: A Systematic Review and an Analysis of the CoLaus Study
Source: PLoS One. 2011 Oct 18;6(10):e26157. doi: 10.1371/journal.pone.0026157 (PMC3196514; doi:10.1371/journal.pone.0026157)
Supplement: Table S5 — Genotype and derived allele frequencies (D) for Q223R, by ethnic group. (DOC) [file pone.0026157.s005.doc]

**Supporting Table S5:** genotype and derived allele frequencies (D) for Q223R, by ethnic group

| **Reference** | **Frequency AA, n (%)** | **Frequency AD, n (%)** | **Frequency DD, n (%)** | **Frequency D, n** | **Frequency D, % (95% CI)** |
| --- | --- | --- | --- | --- | --- |
| **Caucasians** | | | | | |
| Chung 1997 [1] |  |  |  | 88 | 33.59 (27.89-39.66) |
| Silver 1997 [2]^e^ |  |  |  | 181 | 42.89 (38.11-47.77) |
| Silver 1997^f^ | 46 (25.99) | 95 (53.67) | 36 (20.34) | 167 | 47.18 (41.88-52.52) |
| Chagnon 1999 [3] | 95 (30.94) | 146 (47.56) | 66 (21.50) | 278 | 45.28 (41.29-49.31) |
| Oksanen 2000 [4] |  |  |  | 98 | 40.16 (33.96-46.61) |
| Mammes 2001 [5] |  |  |  | 328 | 42.38 (38.87-45.95) |
| Quinton 2001 [6] | 30 (34.09) | 43 (48.86) | 15 (17.05) | 73 | 41.48 (34.11-49.13) |
| Rand 2001 [7]^c^ | 101 (35.82) | 131 (46.45) | 50 (17.73) | 231 | 40.96 (36.87-45.14) |
| Rand 2001^d^ | 59 (34.10) | 76 (43.93) | 38 (21.97) | 152 | 43.93 (38.63-49.34) |
| Wauters 2001 [8] | 77 (27.50) | 139 (49.64) | 64 (22.86) | 267 | 47.68 (43.47-51.91) |
| Yiannakouris 2001 [9] | 52 (44.07) | 56 (47.46) | 10 (8.47) | 76 | 32.20 (26.29-38.57) |
| Mattevi 2002 [10]^a^ | 64 (35.16) | 91 (50.00) | 27 (14.84) | 145 | 39.84 (34.77-45.07) |
| Mattevi 2002^b^ | 47 (30.72) | 86 (56.21) | 20 (13.07) | 126 | 41.18 (35.61-46.92) |
| Van Rossum 2002 [11]^a^ | 94 (30.32) | 152 (49.03) | 64 (20.65) | 280 | 45.16 (41.19-49.17) |
| Van Rossum 2002^b^ | 90 (33.09) | 129 (47.43) | 53 (19.48) | 235 | 43.20 (38.99-47.48) |
| Skibola 2004 [12] | 226 (28.14) | 379 (47.20) | 198 (24.66) | 775 | 48.26 (45.79-50.73) |
| Willett 2005 [13] | 234 (31.03) | 387 (51.33) | 133 (17.64) | 653 | 43.30 (40.78-45.85) |
| Banerjee 2006 [14] |  |  |  | 78 | 41.49 (34.37-48.89) |
| Crabbe 2006 [15] | 74 (27.40) | 145 (53.70) | 51 (18.90) | 247 | 45.74 (41.48-50.05) |
| Portolés 2006 [16] | 379 (43.92) | 381 (44.15) | 103 (11.94) | 587 | 34.01 (31.77-36.30) |
| Snoussi 2006 [17] | 102 (46.00) | 90 (40.50) | 30 (13.50) | 150 | 33.78 (29.39-38.39) |
| De Krom 2007 [18] | 92 (26.74) | 169 (49.13) | 83 (24.13) | 335 | 48.69 (44.90-52.50) |
| Fairbrother 2007 [19] | 336 (30.60) | 536 (48.80) | 226 (20.60) | 988 | 44.99 (42.90-47.10) |
| Mergen 2007 [20] | 157 (39.25) | 202 (50.50) | 41 (10.25) | 284 | 35.50 (32.18-38.93) |
| Podolsky 2007 [21] | 69 (37.00) | 78 (42.00) | 39 (21.00) | 156 | 41.94 (36.87-47.13) |
| Popko 2007 [22] | 26 (33.80) | 40 (51.90) | 11 (14.30) | 62 | 40.26 (32.45-48.46) |
| Richert 2007 [23] | 110 (49.80) | 90 (40.40) | 22 (9.80) | 134 | 30.18 (25.94-34.68) |
| Zhang 2007 [24] | 226 (35.87) | 304 (48.25) | 100 (15.87) | 504 | 40.00 (37.28-42.77) |
| Bienertova 2008 [25] | 51 (27.57) | 94 (50.81) | 40 (21.62) | 174 | 47.03 (41.85-52.25) |
| Den Hoed 2008 [26] | 27 (26.20) | 47 (45.60) | 29 (28.20) | 105 | 50.97 (43.93-57.98) |
| Doecke 2008 [27] | 419 (31.00) | 663 (49.00) | 270 (20.00) | 1203 | 44.49 (42.60-46.39) |
| Iciek 2008 [28] | 4 (12.00) | 21 (62.00) | 9 (26.00) | 39 | 57.35 (44.77-69.28) |
| Masuo 2008 [29] | 43 (33.33) | 55 (42.64) | 31 (24.03) | 117 | 45.35 (39.16-51.64) |
| Marti 2009 [30] | 61 (47.29) | 52 (40.31) | 16 (12.40) | 84 | 32.56 (26.88-38.65) |
| Ragin 2009 [31] | 125 (14.16) | 423 (47.90) | 335 (37.94) | 1093 | 61.89 (59.58-64.16) |
| Szczepankiewicz 2009 [32] | 22 (18.33) | 68 (55.67) | 30 (25.00) | 128 | 53.33 (46.81-59.78) |
| Vasku 2009 [33] | 34 (34.00) | 45 (45.00) | 21 (21.00) | 87 | 43.50 (36.52-50.67) |
| *Heterogeneity* |  |  |  |  | *Q*=517.21, p<0.001* |
| **Asians** | | | | | |
| Chung 1997 [1] |  |  |  | 2 | 50.00 (0.07-93.24) |
| Matsuoka 1997 [34] |  |  |  | 114 | 83.82 (76.54-89.58) |
| De Silva 1999 [35] | 0 (0.00) | 52 (22.41) | 180 (77.59) | 412 | 88.79 (85.56-91.52) |
| Endo 2000 [36] | 15 (2.71) | 134 (24.23) | 404 (73.06) | 942 | 85.17 (82.94-87.22) |
| Koh 2002 [37] | 3 (1.37) | 58 (26.48) | 158 (72.15) | 374 | 85.39 (81.73-88.56) |
| Huang 2003 [38] | 1 (1.28) | 13 (16.67) | 64 (82.05) | 141 | 90.38 (84.68-94.52) |
| Kagawa 2003 [39]^g^ | 0 (0.00) | 5 (10.00) | 45 (90.00) | 95 | 95.00 (88.72-98.36) |
| Kagawa 2003^h^ | 0 (0.00) | 6 (12.00) | 44 (88.00) | 94 | 94.00 (87.40-97.77) |
| Kagawa 2003^i^ | 2 (4.00) | 17 (31.00) | 35 (65.00) | 87 | 80.56 (71.83-87.54) |
| Kagawa 2003^j^ | 1 (2.00) | 17 (27.00) | 45 (71.00) | 107 | 84.92 (77.46-90.67) |
| Kagawa 2003^k^ | 3 (3.00) | 34 (32.00) | 69 (65.00) | 172 | 81.13 (75.21-86.17) |
| Kagawa 2003^l^ | 4 (4.00) | 20 (19.00) | 82 (77.00) | 184 | 86.79 (81.48-91.04) |
| Takahashi 2003 [40] | 8 (3.98) | 39 (19.4) | 154 (76.62) | 347 | 86.32 (82.57-89.52) |
| Ogawa 2004 [41]^a^ | 3 (3.33) | 22 (24.45) | 65 (72.22) | 152 | 84.44 (78.31-89.41) |
| Ogawa 2004^b^ | 4 (3.15) | 25 (19.68) | 98 (77.17) | 221 | 87.01 (82.24-90.89) |
| Wang 2006 [42] | 339 (88.28) | 42 (10.94) | 3 (0.78) | 48 | 6.25 (4.64-8.20) |
| Woo 2006 [43] | 0 (0.00) | 8 (17.80) | 37 (82.20) | 82 | 91.11 (83.23-96.08) |
| Han C.Z. 2008 [44] | 12 (2.37) | 78 (15.60) | 410 (82.03) | 898 | 89.84 (87.76-91.61) |
| Han H.R. 2008 [45] | 8 (2.30) | 69 (20.10) | 266 (77.60) | 601 | 87.61 (84.91-89.98) |
| Kim 2008 [46] | 7 (2.66) | 52 (19.77) | 204 (77.57) | 460 | 87.45 (84.31-90.16) |
| Popruk 2008 [47] |  |  |  | 221 | 86.33 (81.50-90.29) |
| *Heterogeneity* |  |  |  |  | *Q*=373.53, p<0.001* |
| **African-Americans** | | | | | |
| Considine 1996 [48] | 1 (6.67) | 11 (73.33) | 3 (20.00) | 17 | 56.67 (37.43-74.54) |
| Chung 1997 [1] |  |  |  | 17 | 38.64 (24.36-54.50) |
| Podolsky 2007 [21] | 36 (21.00) | 82 (49.00) | 50 (30.00) | 182 | 54.17 (48.67-59.59) |
| Ragin 2009 [31]^m^ | 55 (19.71) | 155 (55.56) | 69 (24.73) | 293 | 52.51 (48.27-56.72) |
| Ragin 2009^n^ | 12 (33.33) | 18 (50.00) | 6 (16.67) | 30 | 41.67 (30.15-53.89) |
| Ragin 2009^o^ | 68 (35.05) | 100 (51.55) | 26 (13.40) | 152 | 39.18 (34.29-44.23) |
| *Heterogeneity* |  |  |  |  | *Q*=21.56, p=0.001* |
| **Mixed populations** | | | | | |
| Chung 1997 [1] |  |  |  | 29 | 37.18 (26.50-48.87) |
| Guízar-Mendoza 2005 [49] | 42 (40.78) | 54 (52.43) | 7 (6.80) | 68 | 33.01 (26.63-39.88) |
| Roth 2005 [50] | 20 (38.00) | 22 (42.00) | 10 (19.00) | 42 | 40.38 (30.87-50.46) |
| Méndez-Sánchez 2006 [51] | 15 (34.90) | 21 (48.80) | 7 (16.30) | 35 | 40.70 (30.22-51.83) |
| Duarte 2007 [52] | 109 (31.14) | 191 (54.57) | 50 (14.29) | 291 | 41.57 (37.89-45.32) |
| Ragin 2009 [31] | 9 (34.62) | 13 (50.00) | 4 (15.38) | 21 | 40.38 (27.01-54.90) |
| *Heterogeneity* |  |  |  |  | *Q*=4.53, p=0.476* |

* Q = Cochran’s Q statistic of heterogeneity

^a^ women

^b^ men

^c^ UK

^d^ India, Bangladesh, Pakistan

^e^ Baltimore Longitudinal Study on Aging

^f^ Johns Hopkins University Weight Management Center

^g^ Japanese men

^h^ Japanese women

^i^ Palauan men

^j^ Palauan women

^k^ Thai men

^l^ Thai women

^m^ Africa

^n^ USA

^o^ Caribbean Islands

**References**

1. Chung WK, Power-Kehoe L, Chua M, Chu F, Aronne L et al. (1997) Exonic and intronic sequence variation in the human leptin receptor gene (LEPR). Diabetes 46: 1509-1511.

2. Silver K, Walston J, Chung WK, Yao F, Parikh VV et al. (1997) The Gln223Arg and Lys656Asn polymorphisms in the human leptin receptor do not associate with traits related to obesity. Diabetes 46: 1898-1900.

3. Chagnon YC, Chung WK, Perusse L, Chagnon M, Leibel RL et al. (1999) Linkages and associations between the leptin receptor (LEPR) gene and human body composition in the Quebec Family Study. International Journal of Obesity & Related Metabolic Disorders: Journal of the International Association for the Study of Obesity 23: 278-286.

4. Oksanen L, Tiitinen A, Kaprio J, Koistinen HA, Karonen S et al. (2000) No evidence for mutations of the leptin or leptin receptor genes in women with polycystic ovary syndrome. Mol Hum Reprod 6: 873-876.

5. Mammes O, Aubert R, Betoulle D, Pean F, Herbeth B et al. (2001) LEPR gene polymorphisms: associations with overweight, fat mass and response to diet in women. Eur J Clin Invest 31: 398-404.

6. Quinton ND, Lee AJ, Ross RJ, Eastell R, Blakemore AI (2001) A single nucleotide polymorphism (SNP) in the leptin receptor is associated with BMI, fat mass and leptin levels in postmenopausal Caucasian women. Hum Genet 108: 233-236.

7. Rand L, Winchester EC, Millwood IY, Penny MA, Kessling AM (2001) Maternal leptin receptor gene variant Gln223Arg is not associated with variation in birth weight or maternal body mass index in UK and South Asian populations. International Journal of Obesity & Related Metabolic Disorders: Journal of the International Association for the Study of Obesity 25: 753-755.

8. Wauters M, Mertens I, Chagnon M, Rankinen T, Considine RV et al. (2001) Polymorphisms in the leptin receptor gene, body composition and fat distribution in overweight and obese women. International Journal of Obesity & Related Metabolic Disorders: Journal of the International Association for the Study of Obesity 25: 714-720.

9. Yiannakouris N, Yannakoulia M, Melistas L, Chan JL, Klimis-Zacas D et al. (2001) The Q223R polymorphism of the leptin receptor gene is significantly associated with obesity and predicts a small percentage of body weight and body composition variability. Journal of Clinical Endocrinology & Metabolism 86: 4434-4439.

10. Mattevi VS, Zembrzuski VM, Hutz MH (2002) Association analysis of genes involved in the leptin-signaling pathway with obesity in Brazil. International Journal of Obesity & Related Metabolic Disorders: Journal of the International Association for the Study of Obesity 26: 1179-1185.

11. van Rossum CT, Hoebee B, Seidell JC, Bouchard C, van Baak MA et al. (2002) Genetic factors as predictors of weight gain in young adult Dutch men and women. International Journal of Obesity & Related Metabolic Disorders: Journal of the International Association for the Study of Obesity 26: 517-528.

12. Skibola CF, Holly EA, Forrest MS, Hubbard A, Bracci PM et al. (2004) Body mass index, leptin and leptin receptor polymorphisms, and non-hodgkin lymphoma. Cancer Epidemiology, Biomarkers & Prevention 13: 779-786.

13. Willett EV, Skibola CF, Adamson P, Skibola DR, Morgan GJ et al. (2005) Non-Hodgkin's lymphoma, obesity and energy homeostasis polymorphisms. Br J Cancer 93: 811-816.

14. Banerjee I, Trueman JA, Hall CM, Price DA, Patel L et al. (2006) Phenotypic variation in constitutional delay of growth and puberty: relationship to specific leptin and leptin receptor gene polymorphisms. Eur J Endocrinol 155: 121-126.

15. Crabbe P, Goemaere S, Zmierczak H, Van P, I, De Bacquer D et al. (2006) Are serum leptin and the Gln223Arg polymorphism of the leptin receptor determinants of bone homeostasis in elderly men? Eur J Endocrinol 154: 707-714.

16. Portoles O, Sorli JV, Frances F, Coltell O, Gonzalez JI et al. (2006) Effect of genetic variation in the leptin gene promoter and the leptin receptor gene on obesity risk in a population-based case-control study in Spain. Eur J Epidemiol 21: 605-612.

17. Snoussi K, Strosberg AD, Bouaouina N, Ben Ahmed S, Helal AN et al. (2006) Leptin and leptin receptor polymorphisms are associated with increased risk and poor prognosis of breast carcinoma. Bmc Cancer 6.

18. de Krom M, van der Schouw YT, Hendriks J, Ophoff RA, van Gils CH et al. (2007) Common genetic variations in CCK, leptin, and leptin receptor genes are associated with specific human eating patterns. Diabetes 56: 276-280.

19. Fairbrother UL, Tanko LB, Walley AJ, Christiansen C, Froguel P et al. (2007) Leptin receptor genotype at Gln223Arg is associated with body composition, BMD, and vertebral fracture in postmenopausal Danish women. Journal of Bone & Mineral Research 22: 544-550.

20. Mergen H, Karaaslan C, Mergen M, Deniz OE, Ozata M (2007) LEPR, ADBR3, IRS-1 and 5-HTT genes polymorphisms do not associate with obesity. Endocr J 54: 89-94.

21. Podolsky RH, Barbeau P, Kang HS, Zhu H, Treiber FA et al. (2007) Candidate genes and growth curves for adiposity in African- and European-American youth. Int J Obes 31: 1491-1499.

22. Popko K, Gorska E, Wasik M, Stoklosa A, Plywaczewski R et al. (2007) Frequency of distribution of leptin receptor gene polymorphism in obstructive sleep apnea patients. Journal of Physiology & Pharmacology 58: 551-561.

23. Richert L, Chevalley T, Manen D, Bonjour JP, Rizzoli R et al. (2007) Bone mass in prepubertal boys is associated with a Gln223Arg amino acid substitution in the leptin receptor. Journal of Clinical Endocrinology & Metabolism 92: 4380-4386.

24. Zhang YY, Gottardo L, Mlynarski W, Frazier W, Nolan D et al. (2007) Genetic variability at the leptin receptor (LEPR) locus is a determinant of plasma fibrinogen and C-reactive protein levels. Atherosclerosis 191: 121-127.

25. Bienertova-Vasku J, Bienert P, Tomandl J, Forejt M, Vavrina M et al. (2008) No association of defined variability in leptin, leptin receptor, adiponectin, proopiomelanocortin and ghrelin gene with food preferences in the Czech population. Nutr Neurosci 11: 2-8.

26. den Hoed M, Smeets AJ, Veldhorst MA, Nieuwenhuizen AG, Bouwman FG et al. (2008) SNP analyses of postprandial responses in (an)orexigenic hormones and feelings of hunger reveal long-term physiological adaptations to facilitate homeostasis. Int J Obes (Lond) 32: 1790-1798.

27. Doecke JD, Zhao ZZ, Stark MS, Green AC, Hayward NK et al. (2008) Single nucleotide polymorphisms in obesity-related genes and the risk of esophageal cancers. Cancer Epidemiology, Biomarkers & Prevention 17: 1007-1012.

28. Iciek R, Wender-Ozegowska E, Seremak-Mrozikiewicz A, Drews K, Sodowski K et al. (2008) Leptin gene, leptin gene polymorphisms and body weight in pregnant women with diabetes mellitus type I. Journal of Physiology & Pharmacology 59: Suppl-31.

29. Masuo K, Straznicky NE, Lambert GW, Katsuya T, Sugimoto K et al. (2008) Leptin-receptor polymorphisms relate to obesity through blunted leptin-mediated sympathetic nerve activation in a Caucasian male population.[see comment]. Hypertension Research - Clinical & Experimental 31: 1093-1100.

30. Marti A, Santos JL, Gratacos M, Moreno-Aliaga MJ, Maiz A et al. (2009) Association between leptin receptor (LEPR) and brain-derived neurotrophic factor (BDNF) gene variants and obesity: a case-control study. Nutr Neurosci 12: 183-188.

31. Ragin CC, Dallal C, Okobia M, Modugno F, Chen J et al. (2009) Leptin levels and leptin receptor polymorphism frequency in healthy populations. Infect Agents Cancer 4.

32. Szczepankiewicz A, Breborowicz A, Sobkowiak P, Popiel A (2009) Are genes associated with energy metabolism important in asthma and BMI? J Asthma 46: 53-58.

33. Vasku A, Vokurka J, Bienertova-Vasku J (2009) Obesity-related genes variability in Czech patients with sporadic colorectal cancer: preliminary results. Int J Colorectal Dis 24: 289-294.

34. Matsuoka N, Ogawa Y, Hosoda K, Matsuda J, Masuzaki H et al. (1997) Human leptin receptor gene in obese Japanese subjects: evidence against either obesity-causing mutations or association of sequence variants with obesity. Diabetologia 40: 1204-1210.

35. de Silva AM, Walder KR, Aitman TJ, Gotoda T, Goldstone AP et al. (1999) Combination of polymorphisms in OB-R and the OB gene associated with insulin resistance in Nauruan males. International Journal of Obesity & Related Metabolic Disorders: Journal of the International Association for the Study of Obesity 23: 816-822.

36. Endo K, Yanagi H, Hirano C, Hamaguchi H, Tsuchiya S et al. (2000) Association of Trp64Arg polymorphism of the beta3-adrenergic receptor gene and no association of Gln223Arg polymorphism of the leptin receptor gene in Japanese schoolchildren with obesity. International Journal of Obesity & Related Metabolic Disorders: Journal of the International Association for the Study of Obesity 24: 443-449.

37. Koh JM, Kim DJ, Hong JS, Park JY, Lee KU et al. (2002) Estrogen receptor alpha gene polymorphisms Pvu II and Xba I influence association between leptin receptor gene polymorphism (Gln223Arg) and bone mineral density in young men. Eur J Endocrinol 147: 777-783.

38. Huang R, Huang XZ, Li M, Xiao Y, Zhang J (2003) [An investigation of the relationship between Lepr gene Gln223Arg polymorphism and obstructive sleep apnea hypopnea syndrome]. [Chinese]. Chung-Hua Chieh Ho Ho Hu Hsi Tsa Chih Chinese Journal of Tuberculosis & Respiratory Diseases 26: 517-521.

39. Kagawa Y, Dever GJ, Otto CT, Charupoonphol P, Supannatas S et al. (2003) Single nucleotide polymorphism and lifestyle-related diseases in the Asia-Pacific region: comparative study in Okinawa, Palau and Thailand. Asia Pac J Public Health 15 Suppl: S10-S14.

40. Takahashi-Yasuno A, Masuzaki H, Miyawaki T, Ogawa Y, Matsuoka N et al. (2003) Leptin receptor polymorphism is associated with serum lipid levels and impairment of cholesterol lowering effect by simvastatin in Japanese men. Diabetes Research & Clinical Practice 62: 169-175.

41. Ogawa T, Hirose H, Yamamoto Y, Nishikai K, Miyashita K et al. (2004) Relationships between serum soluble leptin receptor level and serum leptin and adiponectin levels, insulin resistance index, lipid profile, and leptin receptor gene polymorphisms in the Japanese population. Metabolism: Clinical & Experimental 53: 879-885.

42. Wang TN, Huang MC, Chang WT, Ko AM, Tsai EM et al. (2006) G-2548A polymorphism of the leptin gene is correlated with extreme obesity in Taiwanese aborigines. Obesity 14: 183-187.

43. Woo HY, Park H, Ki CS, Park YL, Bae WG (2006) Relationships among serum leptin, leptin receptor gene polymorphisms, and breast cancer in Korea. Cancer Lett 237: 137-142.

44. Han CZ, Du LL, Jing JX, Zhao XW, Tian FG et al. (2008) Associations among lipids, leptin, and leptin receptor gene Gin223Arg polymorphisms and breast cancer in China. Biol Trace Elem Res 126: 38-48.

45. Han HR, Ryu HJ, Cha HS, Go MJ, Ahn Y et al. (2008) Genetic variations in the leptin and leptin receptor genes are associated with type 2 diabetes mellitus and metabolic traits in the Korean female population. Clin Genet 74: 105-115.

46. Kim SM, Kim SH, Lee JR, Jee BC, Ku SY et al. (2008) Association of leptin receptor polymorphisms Lys109Arg and Gln223Arg with serum leptin profile and bone mineral density in Korean women. Am J Obstet Gynecol 198: 421-428.

47. Popruk S, Tungtrongchitr R, Petmitr S, Pongpaew P, Harnroongroj T et al. (2008) Leptin, soluble leptin receptor, lipid profiles, and LEPR gene polymorphisms in Thai children and adolescents. International Journal for Vitamin & Nutrition Research 78: 9-15.

48. Considine RV, Considine EL, Williams CJ, Hyde TM, Caro JF (1996) The hypothalamic leptin receptor in humans: identification of incidental sequence polymorphisms and absence of the db/db mouse and fa/fa rat mutations. Diabetes 45: 992-994.

49. Guizar-Mendoza JM, Amador-Licona N, Flores-Martinez SE, Lopez-Cardona MG, Ahuatzin-Tremary R et al. (2005) Association analysis of the Gln223Arg polymorphism in the human leptin receptor gene, and traits related to obesity in Mexican adolescents.[see comment]. J Hum Hypertens 19: 341-346.

50. Roth MJ, Paltoo DN, Albert PS, Baer DJ, Judd JT et al. (2005) Common leptin receptor polymorphisms do not modify the effect of alcohol ingestion on serum leptin levels in a controlled feeding and alcohol ingestion study. Cancer Epidemiology, Biomarkers & Prevention 14: 1576-1578.

51. Mendez-Sanchez N, Bermejo-Martinez L, Chavez-Tapia NC, Zamora-Valdes D, Sanchez-Lara K et al. (2006) Obesity-related leptin receptor polymorphisms and gallstones disease. Ann Hepatol 5: 97-102.

52. Duarte SF, Francischetti EA, Genelhu VA, Cabello PH, Pimentel MM (2007) Lepr p.Q223r, beta3-ar p.W64r and lep c.-2548G>A gene variants in obese brazilian subjects. Genetics & Molecular Research 6: 1035-1043.
