# Supplementary material for: Association between Variants of the Leptin Receptor Gene (LEPR) and Overweight: A Systematic Review and an Analysis of the CoLaus Study
Source: PLoS One. 2011 Oct 18;6(10):e26157. doi: 10.1371/journal.pone.0026157 (PMC3196514; doi:10.1371/journal.pone.0026157)
Supplement: Table S7 — Genotype and derived allele frequencies (D) for K656N, by ethnic group. (DOC) [file pone.0026157.s007.doc]

**Supporting Table S7:** genotype and derived allele frequencies (D) for K656N, by ethnic group

| **Reference** | **Frequency AA, n (%)** | | **Frequency AD, n (%)** | **Frequency DD, n (%)** | **Frequency D, n** | **Frequency D, % (95% CI)** |
| --- | --- | --- | --- | --- | --- | --- |
| **Caucasians** | | | | | | |
| Chung 1997 [1] |  | |  |  | 44 | 16.79 (12.48-21.88) |
| Silver 1997 [2]^e^ |  | |  |  | 82 | 19.43 (15.76-23.53) |
| Silver 1997^f^ |  | |  |  | 57 | 16.10 (12.43-20.35) |
| Chagnon 1999 [3]^a^ | 58 (61.70) | | 28 (29.79) | 8 (8.51) | 44 | 23.40 (17.55-30.12) |
| Chagnon 1999^b^ | 36 (52.94) | | 26 (38.24) | 6 (8.82) | 38 | 27.94 (20.59-36.28) |
| Chagnon 1999^c^ | 51 (69.86) | | 19 (26.03) | 3 (4.11) | 25 | 17.12 (11.40-24.23) |
| Chagnon 1999^d^ | 52 (71.23) | | 18 (24.66) | 3 (4.11) | 24 | 16.44 (10.83-23.46) |
| Oksanen 2000 [4] |  | |  |  | 36 | 14.75 (10.55-19.84) |
| Mammes 2001 [5] |  | |  |  | 150 | 19.38 (16.65-22.34) |
| Wauters 2001 [6] | 188 (67.14) | | 82 (29.29) | 10 (3.57) | 102 | 18.21 (15.10-21.67) |
| Yiannakouris 2001 [7] | 70 (59.32) | | 40 (33.90) | 8 (6.78) | 56 | 23.73 (18.45-29.68) |
| Van Rossum 2002 [8]^g^ | 208 (67.10) | | 92 (29.68) | 10 (3.22) | 112 | 18.06 (15.11-21.32) |
| Van Rossum 2002^h^ | 173 (63.60) | | 88 (32.35) | 11 (4.05) | 110 | 20.22 (16.92-23.85) |
| Fairbrother 2007 [9] |  | |  |  | 404 | 15.99 (14.58-17.48) |
| De Luis 2008 [10] | 143 (61.90) | | 81 (30.70) | 7 (7.40) | 95 | 20.56 (16.97-24.54) |
| Doecke 2008 [11] | 933 (69.00) | | 378 (28.00) | 41 (3.00) | 460 | 17.01 (15.61-18.48) |
| Masuo 2008 [12] | 53 (41.09) | | 54 (41.86) | 22 (17.06) | 98 | 37.98 (32.04-44.21) |
| Marti 2009 [13] | 105 (72.41) | | 36 (24.38) | 4 (2.76) | 44 | 15.17 (11.25-19.83) |
| *Heterogeneity* |  | |  |  |  | *Q*= 117,65, p<0.01* |
| **Africans** | | | | | | |
| Chung 1997 [1] |  | |  |  | 5 | 11.36 (3.79-24.56) |
| **Asians** | | | | | | |
| Chung 1997 [1] |  | |  |  | 0 | 0.00 (0.00-0.60) |
| Matsuoka 1997 [14] |  | |  |  | 19 | 13.97 (8.63-20.95) |
| Kagawa 2003 [15]^i^ | 48 (96.00) | | 2 (4.00) | 0 (0.00) | 2 | 2.00 (0.24-7.04) |
| Kagawa2003^j^ | 47 (94.00) | | 3 (6.00) | 0 (0.00) | 3 | 3.00 (0.62-8.52) |
| Kagawa 2003^k^ | 53 (98.00) | | 1 (2.00) | 0 (0.00) | 1 | 0.93 (0.02-5.05) |
| Kagawa 2003^l^ | 63 (100.00) | | 0 (0.00) | 0 (0.00) | 0 | 0.00 (0.00-2.89) |
| Kagawa 2003^m^ | 92 (87.00) | | 12 (11.00) | 2 (2.00) | 16 | 7.55 (4.38-11.97) |
| Kagawa 2003^n^ | 100 (94.00) | | 6 (6.00) | 0 (0.00) | 6 | 2.83 (1.05-6.06) |
| Woo 2006 [16] | 39 (86.70) | | 6 (13.30) | 0 (0.00) | 6 | 6.67 (2.49-13.95) |
| Qu 2007 [17] | 538 (89.82) | | 59 (9.85) | 2 (0.33) | 63 | 5.26 (4.06-6.68) |
| Han H.R. 2008 [18] | 305 (88.90) | | 38 (11.10) | 0 (0.00) | 38 | 5.54 (3.95-7.52) |
| Popruk 2008 [19] |  | |  |  | 11 | 4.30 (2.16-7.56) |
| *Heterogeneity* |  | |  |  |  | *Q*= 24.78, p=0.003* |
| **Mixed populations** | | | | | | |
| Chung 1997 [1] | |  |  |  | 12 | 15.38 (8.21-25.33) |
| Roth 2005 [20] | | 35 (74.00) | 12 (26.00) | 0 (0.00) | 12 | 12.77 (6.77-21.24) |
| Méndez-Sánchez 2006 [21] | | 30 (69.80) | 12 (27.90) | 1 (2.30) | 14 | 16.28 (9.20-25.80) |
| *Heterogeneity* | |  |  |  |  | *Q*= 0.38, p=0.827* |

* Q = Cochran’s Q statistic of heterogeneity

^a^ females with BMI<27

^b^ females with BMI=>27

^c^ males, BMI <27

^d^ males with BMI=>27

^e^ Baltimore Longitudinal Study on Aging

^f^ Johns Hopkins University Weight Management Center

^g^ women

^h^ men

^i^ Japanese men

^j^ Japanese women

^k^ Palauan men

^l^ Palauan women

^m^ Thai men

^n^ Thai women

**References**

1. Chung WK, Power-Kehoe L, Chua M, Chu F, Aronne L et al. (1997) Exonic and intronic sequence variation in the human leptin receptor gene (LEPR). Diabetes 46: 1509-1511.

2. Silver K, Walston J, Chung WK, Yao F, Parikh VV et al. (1997) The Gln223Arg and Lys656Asn polymorphisms in the human leptin receptor do not associate with traits related to obesity. Diabetes 46: 1898-1900.

3. Chagnon YC, Chung WK, Perusse L, Chagnon M, Leibel RL et al. (1999) Linkages and associations between the leptin receptor (LEPR) gene and human body composition in the Quebec Family Study. International Journal of Obesity & Related Metabolic Disorders: Journal of the International Association for the Study of Obesity 23: 278-286.

4. Oksanen L, Tiitinen A, Kaprio J, Koistinen HA, Karonen S et al. (2000) No evidence for mutations of the leptin or leptin receptor genes in women with polycystic ovary syndrome. Mol Hum Reprod 6: 873-876.

5. Mammes O, Aubert R, Betoulle D, Pean F, Herbeth B et al. (2001) LEPR gene polymorphisms: associations with overweight, fat mass and response to diet in women. Eur J Clin Invest 31: 398-404.

6. Wauters M, Mertens I, Chagnon M, Rankinen T, Considine RV et al. (2001) Polymorphisms in the leptin receptor gene, body composition and fat distribution in overweight and obese women. International Journal of Obesity & Related Metabolic Disorders: Journal of the International Association for the Study of Obesity 25: 714-720.

7. Yiannakouris N, Yannakoulia M, Melistas L, Chan JL, Klimis-Zacas D et al. (2001) The Q223R polymorphism of the leptin receptor gene is significantly associated with obesity and predicts a small percentage of body weight and body composition variability. Journal of Clinical Endocrinology & Metabolism 86: 4434-4439.

8. van Rossum CT, Hoebee B, Seidell JC, Bouchard C, van Baak MA et al. (2002) Genetic factors as predictors of weight gain in young adult Dutch men and women. International Journal of Obesity & Related Metabolic Disorders: Journal of the International Association for the Study of Obesity 26: 517-528.

9. Fairbrother UL, Tanko LB, Walley AJ, Christiansen C, Froguel P et al. (2007) Leptin receptor genotype at Gln223Arg is associated with body composition, BMD, and vertebral fracture in postmenopausal Danish women. Journal of Bone & Mineral Research 22: 544-550.

10. De Luis DA, Gonzalez SM, Aller R, Izaola O, Conde R (2008) Influence of Lys656Asn polymorphism of the leptin receptor gene on insulin resistance in nondiabetic obese patients. Journal of Diabetes & its Complications 22: 199-204.

11. Doecke JD, Zhao ZZ, Stark MS, Green AC, Hayward NK et al. (2008) Single nucleotide polymorphisms in obesity-related genes and the risk of esophageal cancers. Cancer Epidemiology, Biomarkers & Prevention 17: 1007-1012.

12. Masuo K, Straznicky NE, Lambert GW, Katsuya T, Sugimoto K et al. (2008) Leptin-receptor polymorphisms relate to obesity through blunted leptin-mediated sympathetic nerve activation in a Caucasian male population.[see comment]. Hypertension Research - Clinical & Experimental 31: 1093-1100.

13. Marti A, Santos JL, Gratacos M, Moreno-Aliaga MJ, Maiz A et al. (2009) Association between leptin receptor (LEPR) and brain-derived neurotrophic factor (BDNF) gene variants and obesity: a case-control study. Nutr Neurosci 12: 183-188.

14. Matsuoka N, Ogawa Y, Hosoda K, Matsuda J, Masuzaki H et al. (1997) Human leptin receptor gene in obese Japanese subjects: evidence against either obesity-causing mutations or association of sequence variants with obesity. Diabetologia 40: 1204-1210.

15. Kagawa Y, Dever GJ, Otto CT, Charupoonphol P, Supannatas S et al. (2003) Single nucleotide polymorphism and lifestyle-related diseases in the Asia-Pacific region: comparative study in Okinawa, Palau and Thailand. Asia Pac J Public Health 15 Suppl: S10-S14.

16. Woo HY, Park H, Ki CS, Park YL, Bae WG (2006) Relationships among serum leptin, leptin receptor gene polymorphisms, and breast cancer in Korea. Cancer Lett 237: 137-142.

17. Qu Y, Yang Z, Jin F, Sun L, Zhang C et al. (2007) Analysis of the relationship between three coding polymorphisms in LEPR gene and obesity in northern Chinese. Obes Res Clin Pract 1: 261-266.

18. Han HR, Ryu HJ, Cha HS, Go MJ, Ahn Y et al. (2008) Genetic variations in the leptin and leptin receptor genes are associated with type 2 diabetes mellitus and metabolic traits in the Korean female population. Clin Genet 74: 105-115.

19. Popruk S, Tungtrongchitr R, Petmitr S, Pongpaew P, Harnroongroj T et al. (2008) Leptin, soluble leptin receptor, lipid profiles, and LEPR gene polymorphisms in Thai children and adolescents. International Journal for Vitamin & Nutrition Research 78: 9-15.

20. Roth MJ, Paltoo DN, Albert PS, Baer DJ, Judd JT et al. (2005) Common leptin receptor polymorphisms do not modify the effect of alcohol ingestion on serum leptin levels in a controlled feeding and alcohol ingestion study. Cancer Epidemiology, Biomarkers & Prevention 14: 1576-1578.

21. Mendez-Sanchez N, Bermejo-Martinez L, Chavez-Tapia NC, Zamora-Valdes D, Sanchez-Lara K et al. (2006) Obesity-related leptin receptor polymorphisms and gallstones disease. Ann Hepatol 5: 97-102.
