# Supplementary material for: Association between Variants of the Leptin Receptor Gene (LEPR) and Overweight: A Systematic Review and an Analysis of the CoLaus Study
Source: PLoS One. 2011 Oct 18;6(10):e26157. doi: 10.1371/journal.pone.0026157 (PMC3196514; doi:10.1371/journal.pone.0026157)
Supplement: Table S8 — Association from linear regression models of LEPR variants with different outcomes. (DOC) [file pone.0026157.s008.doc]

**Supporting Table S8:** association from linear regression models of *LEPR* variants with different outcomes

| **Outcome BMI** |  |  |  |  |  | |  | |  | | |  | | |  | | |  |
| --- | --- | --- | --- | --- | --- | --- | --- | --- | --- | --- | --- | --- | --- | --- | --- | --- | --- | --- |
| **SNP** | **Allele minor/major** | **Beta (SE) heterozygote** | **t-value heterozygote** | **Beta (SE) homoz. Min.** | **t-value homoz. minor** | | **P**  **(chi^2^, 2df)** | | **Beta (SE) additive** | | | **t-value**  **additive** | | | **P add (1df)** | | | **P interaction SNP*sex** |
| rs10128072 | G/T | 0.13 (0.14) | 0.93 | -0.11 (0.38) | -0.27 | | 0.61 | | 0.07 (0.12) | | | 0.59 | | | 0.55 | | | 0.09 |
| rs7518849 | G/A | 0.22 (0.18) | 1.24 | 0.28 (1.13) | 0.24 | | 0.45 | | 0.22 (0.17) | | | 1.25 | | | 0.21 | | | 0.95 |
| rs970467 | T/C | -0.03 (0.15) | -0.23 | -1.11 (0.57) | -1.96 | | 0.14 | | -0.14 (0.13) | | | -1.04 | | | 0.30 | | | 0.83 |
| rs10889553 | T/C | 0.46 (0.20) | 2.25 | 1.10 (1.79) | 0.61 | | 0.07 | | 0.46 (0.20) | | | 2.33 | | | 0.02 | | | 0.70 |
| rs10889567* | C/T | 0.20 (0.14) | 1.44 | 0.19 (0.18) | 1.09 | | 0.31 | | 0.11 (0.09) | | | 1.29 | | | 0.20 | | | 0.90 |
| rs1137100** | G/A | 0.03 (0.13) | 0.22 | -0.08 (0.26) | -0.31 | | 0.92 | | -0.01 (0.10) | | | -0.06 | | | 0.95 | | | 0.61 |
| rs3790438 | A/T | -0.21 (0.14) | -1.53 | -0.06 (0.35) | -0.16 | | 0.31 | | -0.15 (0.12) | | | -1.25 | | | 0.21 | | | **0.04** |
| rs9436746 | A/C | 0.22 (0.13) | 1.71 | 0.28 (0.18) | 1.55 | | 0.15 | | 0.16 (0.09) | | | 1.83 | | | 0.07 | | | 0.69 |
| rs2025805 | A/G | -0.29 (0.14) | -2.04 | -0.16 (0.17) | -0.98 | | 0.12 | | -0.09 (0.08) | | | -1.06 | | | 0.29 | | | 0.85 |
| rs1805096 | T/C | -0.03 (0.13) | -0.24 | -0.23 (0.18) | -1.24 | | 0.45 | | -0.09 (0.09) | | | -1.09 | | | 0.28 | | | 0.26 |
| rs9436748 | T/G | -0.20 (0.14) | -1.38 | -0.01 (0.18) | -0.04 | | 0.30 | | -0.03 (0.09) | | | -0.31 | | | 0.76 | | | 0.72 |
| rs7531110 | G/T | 0.13 (0.13) | 1.00 | 0.11 (0.18) | 0.57 | | 0.59 | | 0.07 (0.09) | | | 0.83 | | | 0.41 | | | 0.34 |
| rs10158279 | C/A | 0.11 (0.14) | 0.80 | 0.25 (0.17) | 1.44 | | 0.35 | | 0.12 (0.09) | | | 1.44 | | | 0.15 | | | 0.46 |
| rs11585329 | A/C | 0.10 (0.14) | 0.75 | -0.34 (0.38) | -0.89 | | 0.47 | | 0.01 (0.12) | | | 0.12 | | | 0.90 | | | 0.27 |
| rs3790437*** | C/T | -0.13 (0.13) | -1.01 | -0.03 (0.31) | -0.11 | | 0.60 | | -0.09 (0.11) | | | -0.80 | | | 0.42 | | | **0.04** |
| **Outcome waist circumference** | | | | | | | | | | | | | | | | | | |
| **SNP** | **Allele minor/major** | **Beta (SE) heterozygote** | **t-value heterozygote** | **Beta (SE) homoz. minor** | | **t-value homoz. minor** | | **P**  **(chi^2^, 2df)** | | | **Beta (SE) additive** | | | **t-value**  **additive** | | **P add (1df)** | | **P interaction SNP*sex** |
| rs10128072 | G/T | 0.54 (0.36) | 1.51 | 0.13 (0.99) | | 0.13 | | 0.32 | | | 0.38 (0.30) | | | 1.27 | | 0.20 | | **0.01** |
| rs7518849 | G/A | 0.75 (0.47) | 1.59 | 2.00 (2.94) | | 0.68 | | 0.23 | | | 0.77 (0.45) | | | 1.71 | | 0.09 | | 0.56 |
| rs970467 | T/C | 0.07 (0.39) | 0.19 | -2.25 (1.47) | | -1.53 | | 0.30 | | | -0.17 (0.35) | | | -0.48 | | 0.63 | | 0.94 |
| **rs10889553** | T/C | 1.64 (0.53) | 3.12 | 3.60 (4.65) | | 0.77 | | 0.01 | | | 1.65 (0.51) | | | **3.12** | | **0.001** | | 0.66 |
| rs10889567* | C/T | 0.53 (0.35) | 1.49 | 0.24 (0.46) | | 0.51 | | 0.32 | | | 0.18 (0.23) | | | 0.80 | | 0.43 | | 0.87 |
| rs1137100** | G/A | -0.10 (0.35) | -0.29 | -0.56 (0.69) | | -0.81 | | 0.71 | | | -0.19 (0.27) | | | -0.72 | | 0.47 | | 0.84 |
| rs3790438 | A/T | -0.47 (0.36) | -1.29 | -0.47 (0.92) | | -0.51 | | 0.41 | | | -0.38 (0.30) | | | -1.27 | | 0.21 | | **0.02** |
| rs9436746 | A/C | 0.54 (0.34) | 1.58 | 0.86 (0.46) | | 1.85 | | 0.12 | | | 0.45 (0.22) | | | 2.03 | | 0.04 | | 0.47 |
| rs2025805 | A/G | -0.70 (0.37) | -1.88 | -0.47 (0.43) | | -0.08 | | 0.17 | | | -0.25 (0.22) | | | -1.15 | | 0.25 | | 0.60 |
| rs1805096 | T/C | -0.10 (0.34) | -0.30 | -0.72 (0.48) | | -1.51 | | 0.31 | | | -0.30 (0.22) | | | -1.32 | | 0.19 | | 0.42 |
| rs9436748 | T/G | -0.89 (0.37) | -2.41 | -0.25 (0.47) | | -0.52 | | 0.04 | | | -0.22 (0.23) | | | -0.95 | | 0.34 | | 0.48 |
| rs7531110 | G/T | 0.24 (0.33) | 0.71 | 0.15 (0.48) | | 0.30 | | 0.78 | | | 0.11 (0.22) | | | 0.51 | | 0.61 | | 0.14 |
| rs10158279 | C/A | 0.37 (0.37) | 0.99 | 0.53 (0.44) | | 1.18 | | 0.46 | | | 0.27 (0.22) | | | 1.21 | | 0.23 | | 0.28 |
| rs11585329 | A/C | 0.16 (0.35) | 0.44 | -0.56 (0.99) | | -0.57 | | 0.75 | | | 0.02 (0.30) | | | 0.05 | | 0.96 | | 0.34 |
| rs3790437*** | C/T | -0.22 (0.34) | -0.66 | -0.16 (0.8) | | -0.21 | | 0.80 | | | -0.17 (0.27) | | | -0.60 | | 0.55 | | **0.04** |
| **Outcome fat mass** | | | | | | | | | | | | | | | | | | |
| **SNP** | **Allele minor/major** | **Beta (SE) heterozygote** | **t-value heterozygote** | **Beta (SE) homoz. minor** | | **t-value homoz. minor** | | **P**  **(chi^2^, 2df)** | | **Beta (SE) additive** | | | **t-value**  **additive** | | | **P add (1df)** | | **P interaction SNP*sex** |
| rs10128072 | G/T | 0.27 (0.26) | 1.03 | -0.13 (0.72) | | -0.17 | | 0.57 | | 0.16 (0.22) | | | 0.73 | | | 0.47 | | **0.047** |
| rs7518849 | G/A | 0.63 (0.34) | 1.84 | 1.54 (2.13) | | 0.72 | | 0.14 | | 0.64 (0.33) | | | 1.96 | | | 0.05 | | 0.70 |
| rs970467 | T/C | 0.07 (0.28) | 0.24 | -1.95 (1.06) | | -1.83 | | 0.18 | | -0.15 (0.25) | | | -0.58 | | | 0.57 | | 0.91 |
| rs10889553 | T/C | 0.96 (0.38) | 2.50 | 2.70 (3.37) | | 0.80 | | 0.03 | | 0.98 (0.37) | | | 2.61 | | | 0.01 | | 0.66 |
| rs10889567* | C/T | 0.31 (0.26) | 1.21 | 0.24 (0.34) | | 0.70 | | 0.47 | | 0.15 (0.16) | | | 0.90 | | | 0.37 | | 0.67 |
| rs1137100** | G/A | 0.03 (0.25) | 0.14 | -0.46 (0.50) | | -0.92 | | 0.62 | | -0.10 (0.19) | | | -0.52 | | | 0.60 | | 0.65 |
| rs3790438 | A/T | -0.33 (0.26) | -1.25 | -0.63 (0.67) | | -0.95 | | 0.33 | | -0.33 (0.22) | | | -1.48 | | | 0.14 | | **0.03** |
| rs9436746 | A/C | 0.41 (0.25) | 1.65 | 0.40 (0.34) | | 1.19 | | 0.22 | | 0.24 (0.16) | | | 1.52 | | | 0.13 | | 0.91 |
| rs2025805 | A/G | -0.42 (0.27) | -1.58 | -0.26 (0.32) | | -0.83 | | 0.29 | | -0.14 (0.16) | | | -0.89 | | | 0.37 | | 0.79 |
| rs1805096 | T/C | -0.10 (0.24) | -0.39 | -0.66 (0.35) | | -1.91 | | 0.15 | | -0.27 (0.16) | | | -1.68 | | | 0.09 | | 0.61 |
| rs9436748 | T/G | -0.23 (0.27) | -0.84 | -0.07 (0.34) | | -0.20 | | 0.69 | | -0.06 (0.17) | | | -0.35 | | | 0.73 | | 0.82 |
| rs7531110 | G/T | 0.24 (0.24) | 0.98 | 0.08 (0.35) | | 0.22 | | 0.61 | | 0.09 (0.16) | | | 0.54 | | | 0.59 | | 0.56 |
| rs10158279 | C/A | 0.30 (0.27) | 1.13 | 0.45 (0.32) | | 1.41 | | 0.34 | | 0.23 (0.16) | | | 1.43 | | | 0.15 | | 0.60 |
| rs11585329 | A/C | 0.19 (0.26) | 0.72 | -0.49 (0.72) | | -0.68 | | 0.58 | | 0.05 (0.22) | | | 0.22 | | | 0.83 | | 0.47 |
| rs3790437*** | C/T | -0.19 (0.25) | -0.76 | -0.52 (0.58) | | -0.89 | | 0.55 | | -0.22 (0.20) | | | -1.08 | | | 0.28 | | **0.03** |
| **Outcome leptin levels** | | | | | | | | | | | | | | | | | | |
| **SNP** | **Allele minor/major** | **Beta (SE) heterozygote** | **t-value heterozygote** | **Beta (SE) homoz. minor** | | **t-value homoz. minor** | | **P**  **(chi^2^, 2df)** | | **Beta (SE) additive** | | | **t-value**  **additive** | | **P add (1df)** | | **P interaction SNP*sex** | |
| rs10128072 | G/T | -0.00 (0.02) | -0.01 | -0.12 (0.07) | | -1.83 | | 0.18 | | -0.02 (0.02) | | | -0.99 | | 0.32 | | 0.78 | |
| rs7518849 | G/A | 0.03 (0.03) | 1.09 | 0.12 (0.20) | | 0.63 | | 0.46 | | 0.04 (0.03) | | | 1.22 | | 0.22 | | 0.33 | |
| rs970467 | T/C | 0.00 (0.03) | 0.09 | -0.16 (0.11) | | -1.56 | | 0.29 | | -0.01 (0.02) | | | -0.56 | | 0.58 | | 0.57 | |
| rs10889553 | T/C | 0.05 (0.04) | 1.30 | 0.26 (0.30) | | 0.88 | | 0.29 | | 0.05 (0.03) | | | 1.46 | | 0.14 | | 0.27 | |
| rs10889567* | C/T | -0.01 (0.02) | -0.52 | 0.02 (0.03) | | 0.62 | | 0.55 | | 0.01 (0.02) | | | 0.40 | | 0.69 | | 0.44 | |
| rs1137100** | G/A | -0.03 (0.02) | -1.09 | 0.01 (0.05) | | 0.23 | | 0.50 | | -0.01 (0.02) | | | -0.56 | | 0.57 | | 0.62 | |
| rs3790438 | A/T | -0.02 (0.02) | -0.74 | -0.01 (0.06) | | -0.16 | | 0.76 | | -0.01 (0.02) | | | -0.65 | | 0.52 | | 0.66 | |
| rs9436746 | A/C | 0.02 (0.02) | 0.77 | 0.01 (0.03) | | 0.29 | | 0.74 | | 0.01 (0.02) | | | 0.48 | | 0.63 | | 0.32 | |
| rs2025805 | A/G | -0.03 (0.03) | -1.09 | 0.01 (0.03) | | 0.23 | | 0.34 | | 0.00 (0.01) | | | 0.16 | | 0.88 | | 0.31 | |
| rs1805096 | T/C | -0.00 (0.02) | -0.20 | -0.04 (0.03) | | -1.14 | | 0.50 | | -0.02 (0.02) | | | -0.99 | | 0.32 | | 0.93 | |
| rs9436748 | T/G | -0.02 (0.03) | -0.86 | 0.01 (0.03) | | 0.38 | | 0.46 | | 0.00 (0.02) | | | 0.17 | | 0.87 | | 0.82 | |
| rs7531110 | G/T | 0.02 (0.02) | 0.66 | 0.01 (0.03) | | 0.30 | | 0.80 | | 0.01 (0.02) | | | 0.49 | | 0.62 | | **0.046** | |
| rs10158279 | C/A | 0.03 (0.03) | 1.12 | 0.04 (0.03) | | 1.35 | | 0.36 | | 0.02 (0.02) | | | 1.38 | | 0.17 | | 0.74 | |
| rs11585329 | A/C | 0.02 (0.02) | 0.98 | 0.04 (0.07) | | 0.63 | | 0.54 | | 0.02 (0.02) | | | 1.11 | | 0.27 | | 0.74 | |
| rs3790437*** | C/T | -0.02 (0.02) | -0.69 | -0.00 (0.05) | | -0.01 | | 0.79 | | -0.01 (0.02) | | | -0.51 | | 0.61 | | 0.44 | |

Results are beta-values with standard errors and t-values from linear regression models (general model and additive model) including age, sex, alcohol consumption, smoking, and the first and second principal components, as covariates. For outcomes other than BMI also height is included as covariate. In addition, the result of the interaction with sex is given. Statistically significant results are shown in bold.

* tag of Q223R

** K109R

*** tag of K656N
