# Supplementary material for: Association between Variants of the Leptin Receptor Gene (LEPR) and Overweight: A Systematic Review and an Analysis of the CoLaus Study
Source: PLoS One. 2011 Oct 18;6(10):e26157. doi: 10.1371/journal.pone.0026157 (PMC3196514; doi:10.1371/journal.pone.0026157)
Supplement: Table S9 — Association from linear regression models of LEPR variants with different outcomes showing a significant interaction with sex, stratified by sex. (DOC) [file pone.0026157.s009.doc]

**Supporting Table S9:** association from linear regression models of *LEPR* variants with different outcomes showing a significant interaction with sex, stratified by sex

| **Outcome BMI** | | | | | | | | | | | | | | | | | | | | |
| --- | --- | --- | --- | --- | --- | --- | --- | --- | --- | --- | --- | --- | --- | --- | --- | --- | --- | --- | --- | --- |
| **SNP** | **Sex** | | | **Allele minor/major** | | **Beta (SE) heterozygote** | | **t-value heterozygote** | | **Beta (SE) homoz. Min.** | | **t-value homoz. minor** | | **P**  **(chi^2^, 2df)** | | **Beta (SE) additive** | | **t-value**  **additive** | **P add (1df)** | |
| rs3790438 | Men | | | A/T | | -0.14 (0.18) | | -0.78 | | 0.75 (0.42) | | 1.80 | | 0.11 | | 0.07 (0.15) | | 0.50 | 0.61 | |
| rs3790438 | Women | | | A/T | | -0.29 (0.21) | | -1.36 | | -1.13 (0.58) | | -1.93 | | 0.08 | | -0.37 (0.18) | | -2.09 | 0.037 | |
| rs3790437*** | Men | | | C/T | | -0.19 (0.17) | | -1.14 | | 0.66 (0.38) | | 1.76 | | 0.08 | | 0.04 (0.14) | | 0.29 | 0.78 | |
| rs3790437*** | Women | | | C/T | | -0.08 (0.19) | | -0.40 | | -0.85 (0.49) | | -1.73 | | 0.22 | | -0.20 (0.16) | | -1.27 | 0.20 | |
| **Outcome waist circumference** | | | | | | | | | | | | | | | | | | | | |
| **SNP** | | **Sex** | **Allele minor/major** | | **Beta (SE) heterozygote** | | **t-value heterozygote** | | **Beta (SE) homoz. minor** | | **t-value homoz. minor** | | **P**  **(chi^2^, 2df)** | | **Beta (SE) additive** | | **t-value**  **additive** | | | **P add (1df)** |
| rs10128072 | | Men | G/T | | -0.00 (0.48) | | -0.01 | | 2.73 (1.36) | | 2.01 | | 0.13 | | 0.60 (0.42) | | 1.45 | | | 0.146 |
| rs10128072 | | Women | G/T | | 0.93 (0.52) | | 1.79 | | -2.22 (1.42) | | -1.56 | | 0.04 | | 0.24 (0.44) | | 0.55 | | | 0.58 |
| rs3790438 | | Men | A/T | | -0.34 (0.48) | | -0.70 | | 1.88 (1.12) | | 1.68 | | 0.15 | | 0.20 (0.40) | | 0.49 | | | 0.62 |
| rs3790438 | | Women | A/T | | -0.59 (0.53) | | -1.11 | | -3.61 (1.47) | | -2.45 | | 0.035 | | -0.97 (0.45) | | -2.15 | | | 0.032 |
| rs3790437*** | | Men | C/T | | -0.43 (0.46) | | -0.95 | | 1.69 (1.01) | | 1.67 | | 0.12 | | 0.17 (0.37) | | 0.47 | | | 0.64 |
| rs3790437*** | | Women | C/T | | -0.06 (0.49) | | -0.12 | | -2.36 (1.23) | | -1.91 | | 0.16 | | -0.47 (0.41) | | -1.15 | | | 0.25 |
| **Outcome fat mass** | | | | | | | | | | | | | | | | | | | | |
| **SNP** | | **Sex** | **Allele minor/major** | | **Beta (SE) heterozygote** | | **t-value heterozygote** | | **Beta (SE) homoz. minor** | | **t-value homoz. minor** | | **P**  **(chi^2^, 2df)** | | **Beta (SE) additive** | | **t-value**  **additive** | | | **P add (1df)** |
| rs10128072 | | Men | G/T | | -0.07 (0.32) | | -0.23 | | 1.41 (0.91) | | 1.55 | | 0.28 | | 0.25 (0.28) | | 0.91 | | | 0.36 |
| rs10128072 | | Women | G/T | | 0.50 (0.40) | | 1.25 | | -1.56 (1.09) | | -1.43 | | 0.14 | | 0.08 (0.33) | | 0.23 | | | 0.82 |
| rs3790438 | | Men | A/T | | -0.30 (0.33) | | -0.91 | | 0.91 (0.75) | | 1.20 | | 0.26 | | 0.02 (0.27) | | 0.06 | | | 0.95 |
| rs3790438 | | Women | A/T | | -0.37 (0.40) | | -0.92 | | -2.74 (1.13) | | -2.43 | | 0.04 | | -0.69 (0.35) | | -2.00 | | | 0.045 |
| rs3790437*** | | Men | C/T | | -0.31 (0.31) | | -1.02 | | 0.85 (0.67) | | 1.27 | | 0.21 | | 0.03 (0.24) | | 0.14 | | | 0.89 |
| rs3790437*** | | Women | C/T | | -0.09 (0.37) | | -0.23 | | -2.13 (0.95) | | -2.26 | | 0.08 | | -0.45 (0.31) | | -1.44 | | | 0.15 |
| **Outcome leptin levels** | | | | | | | | | | | | | | | | | | | | |
| **SNP** | | **Sex** | **Allele minor/major** | | **Beta (SE) heterozygote** | | **t-value heterozygote** | | **Beta (SE) homoz. minor** | | **t-value homoz. minor** | | **P**  **(chi^2^, 2df)** | | **Beta (SE) additive** | | **t-value**  **additive** | | | **P add (1df)** |
| rs7531110 | | Men | G/T | | 0.07 (0.03) | | 2.08 | | 0.02 (0.05) | | 0.42 | | 0.11 | | 0.03 (0.02) | | 1.13 | | | 0.26 |
| rs7531110 | | Women | G/T | | -0.03 (0.03) | | -1.03 | | 0.01 (0.04) | | 0.16 | | 0.50 | | -0.01 (0.02) | | -0.28 | | | 0.78 |

Results are beta-values with standard errors and t-values from linear regression models (general model and additive model) including age, alcohol consumption, smoking, and the first and second principal components, as covariates. For outcomes other than BMI height is also included as covariate.

*** tag of K656N
